# Effectiveness of early warning systems in the detection of infectious diseases outbreaks: a systematic review

**DOI:** 10.1186/s12889-022-14625-4

**Published:** 2022-11-29

**Authors:** Rehab Meckawy, David Stuckler, Adityavarman Mehta, Tareq Al-Ahdal, Bradley N. Doebbeling

**Affiliations:** 1 Public Health and Community Medicine Department, Alexandria Faculty of Medicine, Alexandria University, Champollion street, Al Attarin, Alexandria, Egypt; 2 Department of Social and Political Sciences and Dondena Research Center, Bocconi University, Via Roberto Sarfatti, 25, 20100 Milan, MI Italy; 3 University of Leeds, Leeds, LS2 9JT UK; 4 Institute of Global Health (HIGH), Heidelberg University, Neuenheimer feld 130/3, 69120 Heidelberg, Germany; 5 College of Health Solutions, Arizona State University, 975 S. Myrtle Ave, Tempe, AZ USA

**Keywords:** Early warning system, Notification, Alert, Public health

## Abstract

**Background:**

Global pandemics have occurred with increasing frequency over the past decade reflecting the sub-optimum operationalization of surveillance systems handling human health data. Despite the wide array of current surveillance methods, their effectiveness varies with multiple factors. Here, we perform a systematic review of the effectiveness of alternative infectious diseases Early Warning Systems (EWSs) with a focus on the surveillance data collection methods, and taking into consideration feasibility in different settings.

**Methods:**

We searched PubMed and Scopus databases on 21 October 2022. Articles were included if they covered the implementation of an early warning system and evaluated infectious diseases outbreaks that had potential to become pandemics. Of 1669 studies screened, 68 were included in the final sample. We performed quality assessment using an adapted CASP Checklist.

**Results:**

Of the 68 articles included, 42 articles found EWSs successfully functioned independently as surveillance systems for pandemic-wide infectious diseases outbreaks, and 16 studies reported EWSs to have contributing surveillance features through complementary roles. Chief complaints from emergency departments’ data is an effective EWS but it requires standardized formats across hospitals. Centralized Public Health records-based EWSs facilitate information sharing; however, they rely on clinicians’ reporting of cases. Facilitated reporting by remote health settings and rapid alarm transmission are key advantages of Web-based EWSs. Pharmaceutical sales and laboratory results did not prove solo effectiveness. The EWS design combining surveillance data from both health records and staff was very successful. Also, daily surveillance data notification was the most successful and accepted enhancement strategy especially during mass gathering events. Eventually, in Low Middle Income Countries, working to improve and enhance existing systems was more critical than implementing new Syndromic Surveillance approaches.

**Conclusions:**

Our study was able to evaluate the effectiveness of Early Warning Systems in different contexts and resource settings based on the EWSs’ method of data collection. There is consistent evidence that EWSs compiling pre-diagnosis data are more proactive to detect outbreaks. However, the fact that Syndromic Surveillance Systems (SSS) are more proactive than diagnostic disease surveillance should not be taken as an effective clue for outbreaks detection.

**Supplementary Information:**

The online version contains supplementary material available at 10.1186/s12889-022-14625-4.

## Background

The global pandemic of COVID-19 had a profound impact on the health of the public and economy around the world. Global pandemics have occurred with increasing frequency over the past decade, yet the world has missed opportunities to invest in preparedness and surveillance.

The impact of global infectious disease can be reduced by improving the international exchange of information, and developing monitoring and early warning systems [1]. The COVID-19 pandemic and its emerging variants have urged questioning the effectiveness of the Early Warning Systems (EWSs) for detecting infectious disease outbreaks. Although Dr. Li Wenliang, a Chinese ophthalmologist, issued an emergency warning of abnormal pneumonia cases in December 2019, many countries including the US did not respond optimally [2]. The Economist recently built a machine-learning model to estimate the number of excess deaths due to the pandemic for 223 countries [3]. The model estimates that the total number of deaths is 2–4 times higher than the number of confirmed deaths [3]. Estimates of excess deaths due to COVID-19 are from 20 to 25 Million globally [3]. Such losses are attributed to direct virus effect and indirect consequences on health systems’ overburdened capacity in both developed and developing countries [4].

The recent Monkeypox virus spread is a reminder of the need for diligent surveillance data monitoring for adequate containment of outbreaks and timely initiation of response measures, given the uncertainties and global fear of a repeated pandemic calamity that had the impact of COVID-19 [5]. Nevertheless, even with widespread evidence, investment in public health pandemic data systems within the health sector continues to be overlooked by most governments globally [4]. For instance, the director of the Statistics Division of the UN Department of Economic and Social Affairs (UN DESA), Stefan Schweinfest, asserted that the lack of data impede proper estimation of the impact of abnormal health events [4]. A recent study in China identified data source collection, integration and analysis as core components of an effective infectious disease EWS [6]. For example, databases from Emergency Departments (EDs), hospital and public health records, pharmacies, or even laboratories, are serving as approaches to infectious diseases surveillance which would generate alerts initiating public health investigations and response. However, despite the wide array of current surveillance methods, the effectiveness of these indicators and systems varies with multiple factors, including resource availability, the context of diseases or symptoms under surveillance, and other population health and social factors.

There is widespread agreement that global human health is vulnerable to existing or emerging infectious diseases due to the sub-optimum operationalization of surveillance systems handling health informatics data [4]. These health informatics systems rely on various mechanisms ranging from paper- or digital-based systems to gather population data and are not limited to technology only, as it is commonly miscomprehended [7]. Thus, EWSs enable early detection of the peaking of symptoms levels above-threshold before cases surge, or prompt recognition of small clustering of cases before prevailing illness overwhelms health systems.

However, there is no ideal effective surveillance system yet available. Proactive recognition of abnormal health events consistent with early epidemics remains challenging. Here we report a systematic review of the effectiveness of different EWSs’ designs in terms of utilized methods for data collection, taking into consideration feasibility in different resource settings.

To our knowledge, this systematic review is the first to examine surveillance systems in terms of evaluating their effectiveness. We bridge this gap by synthesizing the peer-reviewed literature on EWSs’ strategies and efficacy based on the methods of data collection. We accomplished this by conducting a systematic review and narrative synthesis of published studies on Syndromic and Sentinel Surveillance for infectious diseases, observing patients’ symptoms and confirmed diseases, respectively.

We define surveillance as “the ongoing systematic collection, analysis, and interpretation of health data essential to the planning, implementation, and evaluation of public health practice, closely integrated with the timely dissemination of these data to those who need to know” [8]. “Early Warning Systems, (EWS)” include data-based detection systems using health informatics data and approaches for infectious diseases surveillance.

## Methods

### Search strategy and selection criteria

We conducted a systematic review to identify relevant peer-reviewed articles regarding Early Warning Systems (EWSs) for detecting infectious disease outbreaks. PRISMA guidelines were followed in the reporting of the review [9]. Published articles were searched on the following electronic databases; PubMed and Scopus on October 21, 2022. The search included relevant keywords and word variants for early warning systems and infectious disease outbreaks. [see Additional file 1]. There was no language restriction at the primary databases’ search; however, we applied language restrictions to English in further steps of the PRISMA flowchart. Upon authors’ consensus (RM, DS, AM), the adopted approach allowed us to investigate the available literature in terms of the presence of a considerable number of publications on the topic of interest.

PubMed syntax (“Early warning system” OR EWS OR Alert OR “syndromic surveillance” OR SSS OR “syndromic surveillance system”) AND ((“infectious disease” OR “communicable diseases”) AND (outbreak)).

Scopus syntax ((TITLE-ABS-KEY (“Early warning system”) OR TITLE-ABS-KEY (ews) OR TITLE-ABS-KEY (alert) OR TITLE-ABS-KEY (“syndromic surveillance”) OR TITLE-ABS-KEY (sss) OR TITLE-ABS-KEY (“syndromic surveillance system”))) AND (((TITLE-ABS-KEY (“infectious disease”) OR TITLE-ABS-KEY (“communicable diseases”))) AND (TITLE-ABS-KEY (outbreak))).

### Inclusion/exclusion criteria

To be included in the review, articles had to describe and evaluate an early warning system that is either already implemented or currently being implemented. This includes data-based detection systems (e.g. using data from various sources, such as death registries, social media trends, lab results, data on relevant over the counter (OTC) medication from pharmacies). The infectious disease outcomes were reviewed independently by two authors (RM and DS) to estimate the scope of the article, and include only those focusing on infectious diseases with the potential to become pandemics. For example, articles on facility outbreaks (e.g. such as Intensive Care Units) and school food poisoning were excluded.

**Fig. 1:**
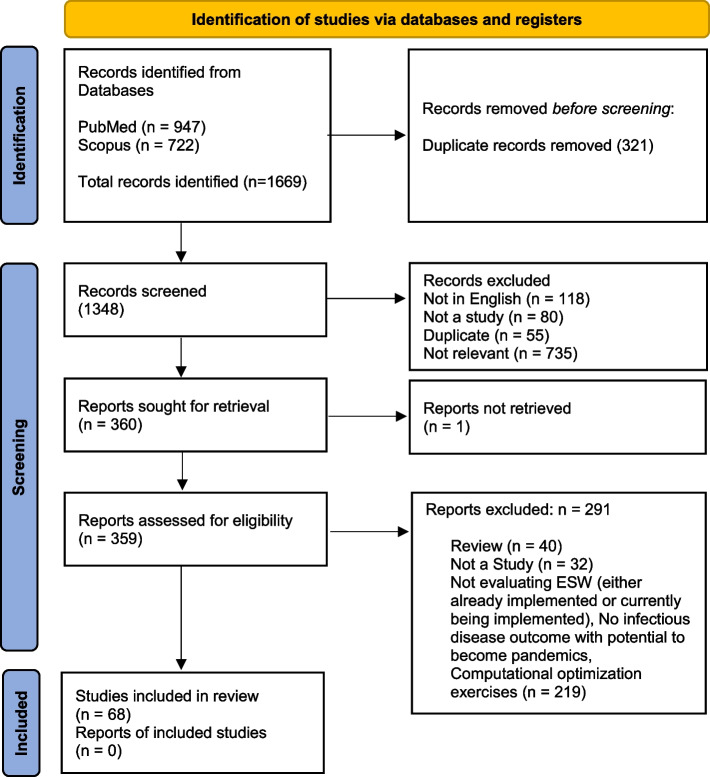
PRISMA flow diagram

We excluded studies that describe projections or proposed EWS which have not yet been evaluated. Articles on computational optimization exercises for outbreaks detection or focusing on non-human outcomes are also excluded. Additionally, commentaries, editorials, correspondences, systematic literature reviews, and preprint articles were also excluded. Eventually, we excluded out-of-date articles – this is because only 2 were detected before 2000, and they used out-of-date technology which is no longer relevant to the current generation of models [10, 11]. Reasons for exclusion are in Fig. 1.

### Data extraction, analysis, and quality assessment

Abstracts and potentially relevant full texts were reviewed independently by two authors (RM and DS) with any conflicts resolved by consensus. The two reviewers screened titles and abstracts, removed duplicates (automatic and manual), and extracted data with the EndNote reference management software.

We assessed the quality of both retrospective and prospective studies for clarity of the research aim and methodology, data reproducibility, comparability, and outcome ascertainment using a 6-question Yes/No questionnaire based on an adapted CASP Checklist and that utilized in evaluating a review on internet-based EWS. Two reviewers (RM and AM) independently assessed the risk of bias and any uncertainty was resolved by contacting the third independent reviewer (DS). An additional file shows the utilized quality assessment tool and the quality assessment results in more detail (see Additional file 2).

We identified the following items for extraction: the author’s name, article’s title, EWS name, country of study, research question, study design, surveilled diseases or manifestations, system’s strengths, weaknesses, recommendations, and effectiveness, as well as comparing systems and study limitations, then used Excel spreadsheets to document all key elements from the included manuscripts.

In some cases, not all of the findings of a study will be relevant to our scope; in such cases, we have included only the relevant findings. Lastly, we extracted a summary of the authors’ interpretations/conclusions. The review was registered with PROSPERO (CRD42021278123) on 18 November 2021 and is reported according to PRISMA guidelines.

## Results

More than half (*n* = 44) of the 68 articles included in the final selection were assessing Early Warning Systems (ESWs) in high-income countries (HICs), including in the US (*n* = 11), Netherlands (*n* = 6), UK (*n* = 5), Australia (*n* = 3), Germany (*n* = 3), Canada (*n* = 3), France (*n* = 2), Italy (*n* = 2), Taiwan (*n* = 2), Japan (*n* = 2), Austria (*n* = 1), New Zealand (*n* = 1), Norway (*n* = 1), Singapore (*n* = 1), Spain (*n* = 1), Korea (*n* = 1). Finally, an additional study, not included in the above calculation, was evaluating EWSs in more than one HICs; including France, Germany, UK, and Spain. On the other hand, 12 studies covered EWSs in middle-income countries (MICs), including China (*n* = 7), India (*n* = 2), Brazil (*n* = 1), Federated States of Micronesia (*n* = 1) and the Republic of Macedonia (*n* = 1). While 10 studies were performed in low-income countries (LICs); Ghana (*n* = 2), Yemen (*n* = 2), Darfur (n-1), Madagascar (*n* = 1), Samoa (*n* = 1), Sierra Leone (*n* = 1), Iraq (*n* = 1) in addition to Pacific island countries and territories (PICTs) (*n* = 1).

All the studies were quantitative, either retrospective (*n* = 47) or prospective (*n* = 15). Three articles assessed the EWS under study both retrospectively and prospectively, while two used the study design of predictive simulation in addition to one cross-sectional study [12]. Table 1 provides a description of included studies with a summary of their key findings. n* is the number of articles evaluating EWSs in different categories; given that some systems were assessed in two articles; NHS Direct syndromic surveillance (*n* = 1 effective + 1 not effective) - ProMED-mail (*n* = 1 effective + 1 limited effective) - National electronic Disease Early Warning System (eDEWS) (*n* = 1 effective + 1 complement) (1 telephone triaging and 1 records)- Electronic Surveillance System for the Early Notification of Community-based Epidemics (ESSENCE) (*n* = 1 effective and 1 not effective but useful) - China Infectious Disease Automated-alert and Response System (CIDARS) (*n* = 2 effective).

**Table 1:** Data Extraction

Author and Year	EWS Name	EWS Category	Purpose/monitored symptoms and diseases	Country	Study design	Outcome reported	Summary of finding	QA Score out of 6
Ansaldi, F.et al.,2009 [13]	Emergency Department Syndrome Surveillance System	ED	5 syndromes including measles-like illness	Italy	Retrospective	Effective	First, the specificity and sensitivity were 94.3 and 91% respectively. Second, the two epidemic threshold breakthroughs anticipated the first notified case by 54 and 11 days. Finally, this was able to anticipate the first case notified by statutory system and virological surveillance to detect signals where low circulation of measles virus was recorded by other systems of surveillance.	6
Cashmore, A. W. et al.,2013 [14]	Public Health Real-Time Emergency Department Surveillance System (PHREDSS)	ED	Pertussis in children	Australia	Retrospective	Not Effective	First, the monitoring of ED visits with cough in children was not able to detect a potential pertussis outbreak before passive surveillance, which includes mandatory reporting from numerous sources including laboratory notification of positive tests. Finally, the RSV activity influences fluctuations in ED visits with cough among the study population.	5
Elliot, A. J. et al.,2012 [15]	Emergency Department Sentinel Syndromic Surveillance System (EDSSS)	ED	Syndromic indicators (respiratory, gastrointestinal, cardiac, acute respiratory infection, gastroenteritis and myocardial ischemia)	United Kingdom	Prospective	Effective	First, this has the potential of identifying severe outbreaks of infectious diseases. Second, the sustainability of the ED-SSS system will continue for several years. Finally, the EDSSS also had the ability to standardize the way data are collected and this can be for multiple uses.	5
Hope, K. G. et al., 2010 [16]	Emergency department syndromic surveillance	ED	Public health conditions during mass gatherings	Australia	Retrospective and Prospective	Effective (Potential)	First, this informs local health action or plays a role as a surveillance safety net for traditional surveillance systems, especially when focusing on pneumonia, meningitis, poisoning and gastrointestinal syndromes. In addition, this has a specific local utility, especially during mass gatherings or disaster response surveillance. Moreover, the ED surveillance data has informed health messages for the media and guided response planning. Finally, this has the potential to detect public health threats that require prompt intervention.	5
Lall, R. et al.,2017 [17]	New York City syndromic surveillance system (The New York City DOHMH Department of Health and Mental Hygiene syndromic surveillance system)	ED	6 use cases (synthetic cannabinoid drug use, heat-related illness, suspected meningococcal disease, medical needs after severe weather, asthma exacerbation after a building collapse, and Ebola-like illness in travelers returning from West Africa	United States	Prospective	Effective	First is the ability to adjust the sensitivity and the specificity, including or excluding keywords or phrases and ICD diagnosis codes. In addition, the surveillance for diseases such as meningitis ED visits helps in tracking unreported cases of culture-negative meningococcal diseases.	6
Muscatello, D. J. et al., 2005 [18]	Public health surveillance system	ED	Syndrome groupings (Abdominal pain Chest pain Convulsions (not clearly epilepsy), Fever (cause unspecified), Collapse/syncope/coma /delirium/ dizziness, Neuromuscular/vision problems, Gastrointestinal syndromes, Cardiovascular syndromes, Respiratory syndromes, Injury syndromes, Other syndromes (Illicit drug-related, Alcohol-related provisional diagnosis, Meningococcal infection, Other or unspecified meningitis Skin problems))	Australia	Retrospective	Effective (Complement)	First is that the ED was able to provide a more timely broad-based surveillance capability than previously available in NSW. However, this doesn’t replace the communicable disease surveillance but worked as a complement for its greater specificity.	4
Terry, W. et al., 2004 [19]	Westchester County’s Syndromic Surveillance System	ED	8 syndrome categories (fever/influenza, respiratory, vomiting, gastrointestinal illness/diarrhea, sepsis, rash, hemorrhagic events, and neurologic)	United States	Retrospective	Effective (Complement)	First, this provides baseline and timely objective data for hospital visits and will be used as a basis for future monitoring of other conditions. Second, several reportable and unusual events during a 9-month period were detected through telephone calls from ED. Finally, this also reflects the importance of disease surveillance communication with local ED staff and indicates the complementary role for syndromic surveillance systems since they are not replacing traditional ones.	6
Tsung-Shu Joseph Wu et al., 2008 [20]	Hospital emergency department-based syndromic surveillance system (ED-SSS)	ED	Fever, respiratory, skin, neurological, upper gastrointestinal (GI), lower GI, haemorrhagic, influenza-like illness (ILI), asthma, enterovirus-related infection (EVI) syndrome, and syndrome for severe illness or death	Taiwan	Retrospective	Effective	First, this can be adapted to other language and cultural environments for improving the global surveillance of infectious diseases. Second, the use of ED-SSS for surveillance of influenza-like illnesses has improved the preparedness of Taiwan’s flu pandemic. Finally, this system was not able to reveal obvious trends in all syndrome groups.	4
Ansaldi, F. et al., 2008 [21]	Emergency Department-based syndromic surveillance system	ED	5 syndromes (Influenza-like illness (ILI), Low-respiratory tract illness (LRTI), not-haemorrhagic gastroenteritis, acute hepatitis, fever-with-rash (maculo-papular or vescicular) syndrome	Italy	Retrospective	Effective (Complement)	The specificity and the sensitivity for this system are 90.3 and 72.9% respectively, and it has the ability to alert the public health institutions 2.5 days before than the local surveillance system.	6
Hong et al., 2022 [22]	Emergency Department-Based Syndromic Surveillance	ED	Predict the number of patients with influenza using the daily number of ED visits due to fever	Korea	Retrospective	Effective	This EWS proved to be feasible for predicting influenza cases 14 days earlier than the routine system through surveilling fever chief complaints from emergency departments.	6
Lukowsky et al., 2022 [23]	–	ED	COVID-like symptoms, influenza-like illnesses (ILIs), and non-influenza ILIs	United States	Retrospective	Effective (Adjunct)	This system was the first to monitor COVID-like symptoms rather than influenza-like illnesses. It has retrospectively suggested that COVID-19 symptoms started in October 2019 (prior to COVID-19 known community transmission).	4
Caudle, J. M. et al., 2009 [24]	Telehealth Ontario toll-free helpline	Telephone	Gastrointestinal Illness	Canada	Retrospective	Effective (Complement)	First, The Telehealth Ontario GI call complaints are a novel, timely and representative data stream that shows promise for integration in the detection of unexpected cases in a real-time syndromic surveillance system. Then, without the need to assimilate ED data from disconnected sources, as a single database, telehealth would provide effective disease surveillance. This type of surveillance can also provide reassurance that an infectious disease outbreak is not occurring. Finally, Telehealth data can serve as proxy measures for ED discharge.	5
Cooper, D. L. et al., 2006 [25]	NHS Direct Surveillance System	Telephone	Syndromes which may represent the prodromal stages of disease caused by a bio-terrorist attack, or more likely a rise in common infections	United Kingdom	Retrospective	Not Effective	First, the NHS Direct surveillance system was most suited for the detection of more widespread rises in syndromes in the community, but at the same time this was unlikely to detect an event like the cryptosporidiosis outbreak. Second, the rise in NHS direct call rates helps in improving the early warning of outbreaks by using call data. Third, when nine-tenths of cases telephoned NHS Direct (using the confidence interval method) there was a one-in-two chance of detection prior to the date officials were notified of this outbreak. Finally, after a substantial rise in call rates there is a full potential for the surveillance system to detect local outbreaks. This work provided information about the nature of syndromic data required to trigger ‘exceedances’.	6
Doroshenko, A. et al., 2005 [26]	NHS Direct syndromic surveillance	Telephone	10 syndromes (cold/influenza, cough, diarrhea, difficulty breathing, double vision, eye problems, lumps, fever, rash, and vomiting)	United Kingdom	Prospective	Effective	First the NHS direct syndromic surveillance has a representative national coverage, It has the potential to detect high-risk, large-scale events by providing near real-time data analysis. Second, this system is useful and acceptable with low marginal costs and borderline portability and flexibility. NHS direct surveillance by the majority of stakeholders was able to detect the national (England and Wales) outbreaks of ILI and increases in diarrhea and vomiting.	6
Dureab, F.et al., 2019 [27]	National electronic Disease Early Warning System (eDEWS)	Telephone	Cholera	Yemen	Retrospective	Effective	This system is sensitive. Second, it can detect and send alerts to health authorities about cholera cases under conditions of ongoing war and civil war, but the timeliness of the response needs improvement. Third, between the first response and reporting the mean time was 2.85 days. Finally, this system is not the only source of data collection of an outbreak of infectious disease, but it is important for the detection of newly emerging cases.	5
Katayama, Y. et al., 2020 [28]	–	Telephone	Seasonal influenza	Japan	Retrospective	Effective	This system showed a positive relationship between the number of telephone triages for fever and the number of patients with influenza in a large metropolitan area in Japan.	5
Katayama, Y. et al., 2021 [29]	–	Telephone	Rotavirus in pediatric patients	Japan	Retrospective	Effective	This showed that the number of pediatric patients with rotavirus incidence was positively related to the number of telephone triage symptoms in a large metropolitan area of Japan. Also, there is a high correlation between traditional surveillance data and the number predicted from the linear regression model, indicating that it would be possible to predict an epidemic of rotavirus earlier than with traditional surveillance.	5
Kavanagh, K. et al., 2012 [30]	Exception reporting system (ERS)	Telephone	Influenza-like illness (ILI)	Scotland	Prospective	Effective	First, this was able to detect exceedances in syndromes related to influenza A (H1N1v) in advance of media reporting. Second, it helps in providing useful information on trends within an area with reassurance when the exceedances are lacking in areas where outbreaks are not known to be occurring. This system is timely with a lag of one reporting day. Finally, during pandemics this system is useful to track temporal changes in the data by eye, but the alarm system is less sensitive unless upward fluctuations occur again.	4
Mostashari, F. et al.,2003 [31]	–	Ambulance	Influenza-like illness (ILI)	United States	Predictive simulation and prospective	Effective	First is the system’s sensitivity for the communitywide outbreaks of the respiratory system with a little negative alarm. Second, this system was able to track epidemics of influenza in the period 1994–1998 and prospectively 1999–2002. Third, the rate of alarms was sustainable with only five alarms not well explained at the 99% level in around 2200 days of surveillance. Fourth, this system was able to detect not only communitywide respiratory outbreaks, but also the ones caused by biologic terrorism. Lastly, the availability of the data is timely and can be categorized into syndromes. Finally, this requires so many nonspecific illnesses to occur before the detection of the event.	4
Schrell, S. et al.,2013 [32]	System for Information on Detection and Analysis of Risks and Threats to Health (SIDARTHa)	Ambulance	Influenza-like illness (ILI)	Spain	Retrospective	Effective (Complement)	First, sensitivity and specificity are 70/63% and 70/63% respectively. Second, is the ability to perform complement sentinel influenza surveillance. Third, the onset of the high influenza in one season was one week before. However, in the other seasons it was at the same time as the sentinel system. Third, in comparison to the weekly sentinel reporting, the daily ED report provides an earlier warning system. Finally, by using the ED Sys system, the beginning of the influenza season in season 2010–11 could be detected in near real time.	5
Ye, C. et al., 2016 [33]	Pudong Syndromic surveillance and Early Warning System (PD-SEWS)	Hospital	7 targeted syndromes (Acute respiratory and Gastrointestinal syndromes, Rash with fever, Neurological syndrome, Hemorrhagic fever, Botulism-like syndrome, Acute viral hepatitis)	China	Prospective	Effective	First, this system is sensitive. Second, this system acted as a practical tool for recording symptoms during the 41st World Exposition in Shanghai in the hospital-based enhanced syndromic surveillance. In addition, each signal which is represented as an aberration was required to be verified timely and investigated by the local staff of Center for Disease Control and Prevention, and as a result, control measures were conducted rapidly, especially when the signal seemed to lead to an outbreak.	6
Buda, S. et al., 2017 [34]	SARI (Severe Acute Respiratory Infections) surveillance system	Hospital	Acute respiratory infections, specifically influenza	Germany	Retrospective	Effective	First, the system has created a SARI baseline to be used as a starting point for the evaluation of severity of future Influenza epidemics or pandemics. Second, the EWS will allow a detailed analysis which focuses on different subgroups of patients with specific risk factors and underlying conditions. In addition, the temporal pattern of hospitalized SARI patients in sentinel data corresponded well to the course of the MAARI incidence along with the virological results of the Germen outpatient sentinel (AGI).	6
Dembek, Z. F. et al., 2004 [35]	Hospital Admissions Syndromic Surveillance statewide syndromic surveillance (HASS)	Hospital	11 syndromes (Pneumonia, hemoptysis, respiratory distress, acute neurologic illness, nontraumatic paralysis, sepsis and nontraumatic shock, fever with rash, fever of unknown cause, acute gastrointestinal illness, and possible cutaneous anthrax, and suspected illness cluster)	United States	Prospective	Effective	First, this system is considered sensitive but not totally. Second, the HASS has been successfully used to identify and investigate individual cases of relatively unusual syndromes (e.g., the detection of two cases of West Nile virus encephalitis by following up on reports of encephalitis in one hospital). However, this system was not able to detect an outbreak that was not detected by other means.	6
Dureab, F. et al., 2020 [36]	National electronic Disease Early Warning System (eDEWS)	Hospital	16 communicable diseases (later increased to 31); respiratory diseases or airborne diseases, digestive system diseases or water/food borne diseases, vector borne diseases, vaccine-preventable diseases, and all other infectious diseases such as chicken pox, brucellosis, schistosomiasis, rabies, HIV/AIDS, tuberculosis (TB), scabies and Guinea worm	Yemen	Retrospective	Effective (Complement)	First, this system is sensitive. Second, this system was the only system in Yemen that provides regular data on communicable diseases. Third, this system was able to detect changes over time since data can be supported by field investigation and laboratory testing. Fourth, this system is very useful in disease trends monitoring in Yemen. Next, this can complement the routine disease surveillance to detect potential outbreaks in a timely way. Finally, there is a challenge in the response timeliness as only 21% of all alerts were verified within the 24 hours in the year 2016.	5
Aggrawal et al., 2020 [37]	–	Hospital	22 acute diseases/syndromes (66% for acute diarrhoeal diseases, 8% for thermal event, 16% for vector-borne diseases, 8% for vaccine-preventable disease)	India	Retrospective and Prospective	Effective	First, this system enabled surveillance systems to respond and monitor risks to public health and detect diseases early. Second, the outbreaks such as gastroenteritis and varicella were confirmed and investigated, and with the implementation of interventions this has prevented additional morbidity and mortality. Finally, the web portal ensured a faster data analysis and interpretation.	5
Ang, B. C. et al., 2005 [38]	Patient Care Enhancement System (PACES)	Hospital	Gastrointestinal (GI), Fever, Respiratory, Coma, Neurological, Dermatologic-Haemorrhagic, Dermatologic-infectious	Singapore	Retrospective	Effective	First, it is sensitive. Second, by utilizing repeat consults, there was a potential to improve the signal-to-noise ratio. This has resolved the level of subunits at risk which can detect sizes of outbreaks that are difficult to achieve with community-based systems.	5
Kool, J. L. et al., 2012 [39]	Syndromic Surveillance System	Hospital	Influenza-like illness (ILI) and laboratory-confirmed influenza	Pacific island countries and territories (PICTs)	Retrospective	Effective (Complement)	First, it doesn’t replace the need for laboratories to report confirmed cases of outbreak-prone diseases such as typhoid fever, dengue, influenza, and leptospirosis. Second, it replaces the routine collection of large amounts of data in cases that this data might not be used for early warning purposes. Third, it enables public health response by control measures by the identification of outbreaks. Finally, it improves communication across borders on infectious diseases	4
Fan, S. et al., 2010 [40]	The Alberta Real Time Syndromic Surveillance Net (ARTSSN)	Public Health Records	3 goals: 1) improve upon traditional paper-based, fragmented public health surveillance using a centralized automated system; 2) enhance routine public health surveillance through effective use of existing Provincial Electronic Health Record data for earlier **detection of cases, clusters, outbreaks and trends of communicable disease, injury, and environmental hazard exposure**; and 3) track the effectiveness of public health interventions	Canada	Prospective	Effective	This system was very helpful in terms of providing richly integrated information on a variety of health conditions for early detection and prompt action on abnormal events such as clusters, outbreaks and trends.	6
Lowe, R. et al., 2016 [41]	Prototype Early Warning System	Public Health Records	Dengue risk	Brazil	Retrospective	Effective	First, this system has the potential to be useful in public health services and its use goes beyond the mass gatherings to control potential explosive dengue epidemics. Second, the forecast model was able to detect extreme dengue incidence rates. Finally, it was able to predict high risk of dengue correctly for 57% of the microregions.	5
Nuvey, F. S. et al., 2019 [42]	Ghana’s ILI surveillance system	Public Health Records	Influenza-like illnesses (ILI)	Ghana	Retrospective	Effective (Partial)	First, this system is sensitive and representative. Second, it has suboptimal data quality. Third, in terms of timeliness, this system takes on average 10 days between the symptom onset and detection at facilities. Next, even though the positive predictive value is low, it generally conforms to other syndromic surveillance systems with broad case definitions aiming at maximizing influenza case detection. In addition, this was able to detect influenza viruses in circulation.	5
Yang, W. et al., 2011 [43]	China Infectious Disease Automated-alert and Response System (CIDARS)	Public Health Records	28 notifiable infectious diseases	China	Retrospective	Effective	First, this assists with early outbreak detection locally and connects reporting of unusual disease occurrences or potential outbreaks to CDCs throughout the country. Second, it helps in shortening the frequency of surveillance data analysis and that of the communication of outbreaks among different CDCs. Third, it reduces the workload of data collection and analyzing for epidemiologists. Finally, this might be less timely and sensitive than other outbreak detection systems that used data on pre-diagnosis of cases in hospitals, media reports or school absenteeism.	6
Zhang, H. et al., 2014 [44]	China Infectious Disease Automated-alert and Response System (CIDARS)	Public Health Records	Dengue Fever, CIDARS automatically conducts the aberration detection from the reported data in the web-based Nationwide Notifiable Infectious Diseases Reporting Information System (NIDRIS).	China	Prospective	Effective	First, this system has a sensitivity and specificity of 100 and 99.8% respectively. Second, it is successful in detecting all DF outbreaks. Third, this is assisting the local public health staff in early detection of potential outbreaks. Next, it is so useful as a decision tool for the control of dengue fever and risk-management programs 5-Median time to detection of 3 days.	4
Baghdadi, Y. et al., 2019 [45]	Reactive mortality surveillance system- syndromic surveillance system SurSaUD	Public Health Records	Combines both morbidity and mortality data (all-cause mortality surveillance system)	France	Retrospective	Effective	First, this system was able to detect weeks with alarm with sensitivity and specificity of 0.96 and 0.99 respectively at the national level and for all ages. Second, it has the potential to follow and detect mortality outbreaks. Third, this is a useful tool in early evaluation of the impacts of threats on mortality and to alert decision makers to adapt control measures. Fourth, it provides weekly information and recommendations in the form of reports for decision makers. Finally, It is useful in reinforcing recommendations for control and prevention measures.	4
Guzman-Herrador, B. et al., 2016 [46]	National web-based outbreak rapid alert system (Vesuv)	Web	(1) outbreaks caused by infectious diseases that are notifiable to MSIS, (2) outbreaks suspected to be associated with food or water, (3) outbreaks of particularly severe illnesses (i.e. diseases with high mortality, high complication rate, or otherwise severe manifestations not otherwise notifiable to MSIS), (4) particularly extensive outbreaks, and (5) outbreaks in healthcare institutions *MSIS: Meldingssystem for smittsomme sykdommer (MSIS) Norwegian Surveillance System for Communicable Diseases	Norway	Prospective	Effective	This system is helpful in terms of enhancing the reporting, and facilitating the sharing, of information between authorities at local and national levels. According to the requirement of international health regulations 2005, it is considered as an important tool for event-based reporting. Finally, it is also enhancing the rapid alerts between stakeholders and the information exchange between different levels of authority.	6
Li, Z. et al., 2014 [47]	China’s infectious disease automated alert and response system	Web	Hand, foot and mouth disease (HFM)	China	Retrospective	Effective	This system has good sensitivity and specificity in the detection of HFM disease outbreaks and has a potential in the reduction of the size of outbreak. Besides, the use of this system continuously is effective in the prevention and reduction of such an outbreak in China. The sensitivity is higher for outbreaks that are large, with more than 20 cases, than for small ones. The overall specificity of the response system was (95.0% CI) and the overall mean time to detection was 2.1 days (95% CI). The mean time of detection for outbreaks with more than 20 cases is 2.7 days while it is only 1.7 days for the smaller outbreaks with less than 10 cases. Finally, the mean time from detection to reporting to the public health emergency system was 4.5 days.	4
Stikova, E. et al., 2010 [48]	An early warning system (ALERT) for priority communicable diseases	Web	Foodborne or waterborne diseases, Acute respiratory syndrome, Acute hemorrhagic fever syndrome, Other zoonotic diseases, Acute neurological syndrome, Vector-borne disease, Vaccine-preventable diseases, Influenza (A/H5 virus), Influenza (novel virus, not H5), Cholera, Yellow fever, Plague, Others	The Republic of Macedonia	Retrospective	Effective (Complement)	First, sensitivity and usefulness of this system should be increased. Second, according to their experience, the reporting units at primary health care levels are not the most appropriate for early detection notification of some epidemic-prone diseases. In addition, adding emergency departments as notification sources for some syndromes, better defining the role of the laboratory to confirm the suspicion of outbreaks, revising the list and definition of syndromes to adjust their sensitivity and specificity for detecting the targeted diseases, and strengthening data analysis through training are necessary.	1
Ganeshkumar et al., 2022 [49]	Digital syndromic surveillance system	Web	17 syndromes; Fever, Fever with cough, Dysentery, Diarrhoea, Bites, Heat related illness, Injuries, Drowning, Conjunctivitis, Cutaneous lesions, Acute Jaundice Syndrome, Botulism like syndrome and Unusual syndrome	India	Retrospective	Effective	This system is highly feasible in LMICs given its adaptability and affordability. Through an open-source software, epidemiologists had real-time access to surveillance data of mass gathering events with prompt reporting to higher authorities.	5
Pinto, A. et al., 2005 [50]	EWS	Internet	Acute flaccid paralysis, Acute jaundice syndrome, Acute respiratory infection, Acute watery diarrhoea, Bloody diarrhoea Injuries, Malaria Neonatal tetanus, Severe malnutrition, Suspected measles, Suspected meningitis, Unexplained fever	Darfur	Retrospective	Effective	First, this system sets up a rumor-verification process to increase the sensitivity and the timeliness of the EWS. This EWS is useful for detecting outbreaks and monitoring the number of consultations that are required to trigger actions. Next, The Crude Mortality Rate (CMR) is a key indicator of an emergency in a crisis setting. However, other indicators should be considered such as surveys, grave counting, and daily interviews. Finally, the computer application developed and installed at the state level was a simple tool to enhance surveillance capacities at the peripheral level, improve timeliness in outbreak detection and to facilitate information exchange and feedback through the production of automatic reports.	5
Zeldenrust, M. E. et al., 2008 [51]	ProMED-mail; the Program for Monitoring Emerging Diseases	Internet	Emerging diseases involving humans, animals and plants around the world	Netherlands	Retrospective	Effective (limited)	First, this is sensitive but not very specific. Second, it has a limited but real added value than the other sources. In addition, ProMed-mail is considered as the only source of information about a VPD outbreak (Vaccine preventable disease).	4
Witkop et al., 2009 [52]	Electronic Surveillance System for the Early Notification of Community-Based Epidemics (ESSENCE) Version II	Internet	Influenza-like illness (ILI)	United States	Retrospective	Not Effective but useful	First, this system has inadequate sensitivity and poor positive predictive value of (71.4%) and (31.8%) respectively. Second, this has a lack of timeliness (1–3-day delay). Third, it is useful in monitoring local influenza seasons and determining syndromic baselines. Finally, this is not the early warning system for an emerging infectious disease and didn’t detect the outbreak soon enough to establish prevention and control measures that might have slowed the spread of the infection.	5
Betancourt, J. A. et al., 2007 [53]	Electronic Surveilance System for the Early Notification of Community-based Epidemics (ESSENCE)	Internet	Syndrome groups: respiratory, upper gastrointestinal (GI), lower GI, fever, hemorrhagic illness, infectious rash, neurological, and coma or sudden death	United States	Retrospective	Effective	First, this system has a high sensitivity and specificity with a range of sensitivity for the syndromic groups of 65.7, and 89% for respiratory disease and GI diseases respectively. In addition, the specificity ranged from 95.5 to 96% for all MTFs. Finally, the data used by the ESSENCE reflects the types of patient visits to these facilities with a high level of accuracy.	6
Carneiro, H. A. and Mylonakis, E., 2009 [54]	Google Flu Trends	Internet	Influenza	United States	Retrospective	Effective	First, this system is sensitive. Second, it has shown great promise as a timely and robust surveillance system. Next, this is best for diseases with higher prevalence, and it performs better in developed countries because for this system to perform well, it needs a large population of Web search users. Moreover, Google flu trends and possibly GT makes it possible to track the infectious diseases activity faster than the conventional systems. Finally, this was able to estimate Influenza levels 1–2 weeks earlier than published CDC reports.	5
Dion, M. et al., 2015 [55]	Global Public Health Intelligence Network (GPHIN)	Internet	Communicable disease outbreaks, potential chemical and radio nuclear hazards	Canada	Retrospective	Effective	First, this has an efficacy in detecting new illnesses. Second, the adoption of big data in the system has increased the capacity to detect international infectious disease outbreaks. In addition to early detection this system has proven to be effective in monitoring. Moreover, what is meant by big data is the large datasets provided by sources such as social media or newspapers which requires a powerful computational method to reveal trends, patterns or the predictive likelihood of events.	4
Samaras, L. et al., 2021 [56]	–	Internet	Measles	The largest countries of Europe (France -Germany - UK -Spain)	Retrospective	Effective	First, measles cases estimation using Google trends helps in producing acceptable results and predicts outbreaks at least two months in advance. Second, the overall prediction model shows statistically significant correlation between measles cases and predictive values. Third, in terms of time, volume and overall spread, measles can be predicted and estimated. Fourth, countries with relatively low impact of measles had major differences and deviation so alternative models were used to improve the results. Fifth, the ECDC publishes a report monthly that includes data on measles 2–3 months prior to the month of the report announcement. Next, the previously stated countries with low activity of measles show less efficient predictions, such as Spain and UK.	6
El-Khatib, Z. et al., 2019 [57]	Syndrome-Based Surveillance System (SbSS) for Infectious Diseases Among Asylum Seekers	Staff	13 syndromes (rash with fever, rash without fever, acute upper respiratory tract infection, acute lower respiratory tract infection, meningitis or encephalitis, fever and bleeding, non-bloody gastroenteritis or watery diarrhea, bloody diarrhea, acute jaundice, skin, soft tissue, or bone abnormalities, acute flaccid paralysis, high fever with no other signs, and unexplained death	Austria	Retrospective	Effective	First, this system has high sensitivity and low specificity. Second, the SbSS is considered as a reliable method for public health surveillance that is used in different contexts. Third, it supports suitable public health responses and timely information. Finally, this system was reliable at identifying and controlling the spread of infectious disease among the asylum-seeking population from September 2015 onward.	5
Flamand, C. et al., 2008 [58]	Syndromic Surveillance System (SOS Medecins)	Staff	Seasonal outbreaks and unusual events	France	Retrospective	Effective (Complement)	First, this system is highly sensitive. Second, this system is used to assess large episodes of illnesses that do not require hospital admissions or an etiological agent identification. Third, this system has advantages such as timeliness and diagnostic specificity. Next, it has many purposes such as disease patterns monitoring to detect outbreaks and providing timely and detailed information to health authorities. Also, it helps in informing the clinicians of the conditions that are prevalent in the community along with supplementing the current infectious disease surveillance. In addition, it has the potential to show the importance of an early signal to increase the GP’s awareness of the need to bring any unusual events to the attention of the surveillance. This reinforces the links between clinicians and institutions in charge of health security. It helps in the estimation of a heat wave’s health impact to provide data to politicians to make decisions. Finally, it reassures health authorities that an outbreak has not occurred because of a public health alert, such as the potentially dangerous food consumption in a large part of the population of that area.	6
Leining et al., 2022 [12]	Syndromic Surveillance	Staff	Runny nose / congestion/ sneezing Achy muscles/joints Anxiety Headache Depression Cough—productive Stomach pain/cramping Sore throat Cough—non-productive Injury/Skin wounds Malaise/Fatigue/Tired Fever Nausea Rash Diarrhea Animal or insect bites Vomiting	United States	Cross-sectional	Effective	Although the system did not detect a specific outbreak, it successfully surveilled prodromal manifestations among Hurricane Harvey evacuees. Efficiency, effectiveness, acceptability and being user-friendly are its key strengths.	6
Jones, N. F. and Marshall, R., 2004 [59]	Electronic general-practitioner-based syndromic surveillance system (GPSURV)	Staff	3 acute infectious-disease syndromes (gastroenteritis, influenza-like illness, and skin and subcutaneous tissue infection)	New Zealand	Retrospective	Effective	First, it monitors the incidence of acute syndromes as successfully as the manual systems by using the standardized clinical-term data from general-practice clinics that are selected. Second, the provision of feedback for reports appears to have a positive effect on data quality but is limited at the same time. Third, the algorithm that was used for the classification of follow-up visits is probably working effectively. Finally, the number of physicians reporting the increase in compliance during the pilot study was higher than number reporting decreases for all conditions.	4
Merali, S. et al., 2020 [60]	Modified Community-based surveillance (CBS)	Staff	Animal-related events, Suspected rabies and other animal bites Unexpected animal deaths, Vaccine-preventable diseases, Suspected measles, Suspected yellow fever, Acute flaccid paralysis, Suspected meningitis, Chickenpox, Foodborne illnesses, Other infectious diseases (Acute hemorrhagic conjunctivitis, Malaria, Skin diseases, Suspected cholera Infectious arthritis, Adverse event following immunization)	Ghana	Retrospective	Effective	First, this is a highly sensitive system. Second, it has an attempt to incorporate a one-health approach at the community level. Third, it is useful in the detection of diverse public health events that might be missed by routine surveillance systems. Fourth, this system was able to detect about 26% of all suspected vaccine-preventable cases from the implementing districts through routine disease surveillance. The one-health approach can address the urgent ongoing or potential health threats at the human animal nexus at sub-national, national, regional, or global levels and this requires balance and collaboration among multiple relevant sectors and disciplines. In addition, both the event-based surveillance and one health are complementary. The one health can enhance the EBS by providing a platform for coordinating surveillance and information sharing across all relevant sectors. It enhances the One-health by rapid detection of a wide range of events between humans, animals and the environment including zoonotic diseases.	3
Murray, K. O. et al., 2009 [61]	Emerging Disease Syndromic Surveillance	Staff	Fever, vomiting, diarrhea, sore throat, cough, runny nose, rash, and communicable respiratory diseases (influenza or common cold)	United States	Prospective	Effective	This was successful in confirming the acute gastroenteritis outbreak and became a critical tool in monitoring the course of the outbreak. During the surveillance period, they were able to identify a steady increase in reporting of respiratory symptoms.	3
Randrianasolo, L. et al., 2010 [62]	Sentinel syndromic-based surveillance system	Staff	Circulating arboviruses (changes in fever frequency during fever-associated diagnoses and changes in diarrheal frequency)	Madagascar	Retrospective	Effective (Complement)	First, this showed the feasibility of implementing syndrome surveillance in a developing country at low cost with good cooperation by SGPs (daily data transfer rate estimated to 89%) and a minimum of effort by staff. Then, this system was able to detect by the use of plot peaks in febrile syndromes, including which disease among influenza, malaria or arboviruses was the most potential cause.	5
Ratnayake, R. et al., 2016 [63]	Community Event–Based Surveillance for Ebola Virus Disease (CEBS)	Staff	Ebola virus disease- CEBS was designed to supplement the national surveillance system by training community members to identify, within their own communities, unsafe burials and persons with signs and symptoms compatible with EVD infection.	Sierra Leone	Retrospective	Effective	First, it is highly sensitive. Second, the CEBS detected cases were more rapidly identified than by the national surveillance system. The CEBS system is capable of quickly finding cases, especially those with no identified epidemiological links. This helps in making the system capable in the detection of early stages of new infectious disease outbreaks or to rapidly identify the spread of disease to new geographic areas. Then, the CEBS was effective in generating alerts for, and was able to detect, a third of all EVD cases found in the districts.	5
Weng, T. C. et al., 2015 [64]	School-Based Syndromic Surveillance System (SID-SSS)	Staff	5 syndrome groups (most common pediatric); enterovirus, influenza, conjunctivitis, diarrhea, and others (chicken pox, scabies, and head louse infection)	Taiwan	Retrospective	Effective (Complement)	First, this system completely replaces the ED-SSS, but the two systems complement each other. Second, it has the ability to detect timely signals for common pediatric infectious diseases. Third, it has a role in being the first line of alert at the beginning stage of pandemics. Through identifying the secondary cases at schools and families (clusters) this has helped in estimating the transmissibility caused by different strains within the same type/subtype/genotype of Enteroviruses of influenza viruses. Enterovirus and influenza-like illnesses were the most reported syndrome groups with (77.6 and 15.8% among a total of 19,334 cases, respectively). The pre-diagnostic data reported from the SID-SSS offered a 1–2-week advance in detecting the peaks of EVI and ILI, indicating a marked improvement from the hospital-based surveillance system.	5
Lai et al., 2021 [65]	Sentinel syndromic surveillance system	Staff	Daily counts of ‘total’ and ‘cough’ absence reports	United Kingdom	Retrospective	Effective (Complement)	This system gathered school absenteeism data which has supported tracking community morbidity such as for COVID-19, and other respiratory and gastrointestinal infections.	5
Yang et al., 2022 [66]	“Xiao Lian Xing” (XLX), SSS based app	Staff versus technology	Daily and weekly absenteeism and fever rates	China	Prospective	Effective with less capacity	This system highlighted that utilization of information technology to record school absenteeism has better “simplicity, cost-effectiveness, data quality, sensitivity, and timeliness metrics over manual approaches (by school staff). (completeness of 100% versus 86.7%, respectively)	6
Groeneveld, G. H. et al., 2017 [67]	ICARES: a real-time automated detection- ICARES (Integrated Crisis Alert and Response System)	Staff	Infectious diseases (respiratory tract infection, hepatitis and encephalitis/meningitis)	Netherlands	Prospective	Effective (Complement)	First, this is a specific system. It is considered as the first flexible automated, real-time cluster detection system for infectious diseases based on information from the hospitals and general practitioners. Then, this system has the ability to detect and follow small regional clusters in real time and can handle any diseases that are registered by first line physicians.	6
Lami et al. 2021 [68]	Syndromic surveillance system	Staff	Common acute and infectious conditions, chronic conditions, and trauma and injuries	Iraq	Prospective	Effective (Complement)	This system provided assistance to health staff to monitor common infectious diseases during mass gathering events where combining fever and cough syndromic surveillance data alerted health staff of the rise in influenza-like illness.	6
Das, D. et al.,2015 [69]	–	Drugs	Influenza-like Illness (cough and influenza medications) and gastrointestinal illness (major brand and generic antidiarrheal)	United States	Retrospective and Prospective	Effective (Adjunct)	First, this is not as sensitive as ED systems. Second, this system has found that the antidiarrheal medication sales for diseases such as Norovirus and influenza were more sensitive than for other illnesses caused by rotavirus. Third, this is more useful in large-scale illness trends. Fourth, for other indications of citywide illness this played a role as an adjunct. In addition, the ILI sales were not able to provide an earlier warning than the ED system of communitywide influenza. Last, GI medications didn’t increase during late winter as ED diarrheal visits. Finally, the sales of citywide OTC medications can provide indications of a communitywide illness such as the annual influenza epidemic.	6
Dong, X.et al.,2017 [70]	Over-The-Counter (OTC) drug sales, Hospital and School-based influenza-like illness (ILI) and Baidu search queries	Drugs	Influenza-like illness (ILI)	China	Retrospective	Effective (Complement)	First, there was a very strong correlation between the research for terms on the Baidu Internet; for example, fever and laboratory-confirmed influenza activity obtained during 2013–14 period of reporting. However, the correlation was moderate among other influenza syndromic surveillance systems. Finally, using this type of data together with traditional laboratory surveillance can provide more timely and sensitive information about current influenza activity.	6
van Benthem, B. H. and van Vliet, J. A., 2008 [71]	The Netherlands’ Infectious diseases Surveillance Information System (ISIS)	Lab	Test results of all microorganisms and Trends in antimicrobial resistance	Netherlands	Prospective	Not Effective	This ISIS system was not considered suitable as an early warning since other systems were better in detecting outbreaks.	4
Bijkerk, P. et al., 2017 [72]	Netherlands Early Warning Committee	Records + Internet	All infectious diseases that can potentially threaten Dutch public health	Netherlands	Retrospective	Effective	First, this system is sensitive. Second, it provides background and preliminary outbreak information. Third, there are only 2% of the potentially relevant threats by using the ECDC RT Report and ProMed-mail. Fourth, they showed that to detect threats internationally by the weekly report, it is enough to screen the ECDC Round Table Report and ProMed-mail. Finally, these ECDC RT reports and ProMED-MAIL were the most complete and timely sources for the identification of infectious diseases threats. This combination between the two sources has resulted in 169 (95%) timely reported threats with only six missed threats and three threats not detected in a timely manner.	4
van den Wijngaard, C. et al., 2008 [73]	Syndromic Surveillance System	Records + Lab	Emerging respiratory diseases	Netherlands	Retrospective	Effective	First, earliest syndrome elevations were observed in absenteeism data, followed by hospital data (+1 week), pharmacy/general practitioner consultations (+2 weeks), and deaths/laboratory submissions (test requests) (+3 weeks). We are confident in concluding that the GP, hospital, laboratory submission, and mortality syndromes do reflect pathogen activity sufficiently for use in syndromic surveillance. Second, using seasonal terms as an addition, they observed that for the absenteeism and, to a lesser extent, the pharmacy registry, the associations between the respiratory syndromes and the pathogen counts might be biased to some extent. Finally, they find that using data from multiple registries together has an advantage so that signal detection can be made more specific by focusing on signals that occur concurrently in > 1 data source.	4
van den Wijngaard, C. C.et al., 2010 [74]	–	Records + Staff	Lower Respiratory Infections	Netherlands	Predictive simulation	Effective (Complement)	First, the sensitivity for local outbreaks was reduced by using data with relatively low coverage levels. Methods other than space-time statistics are more appropriate to generate useful information for such data sources with low coverage in the public health practice. Second, this can detect local LRI-outbreaks in a timely manner. Third, Internet based ILI monitoring along with virological self-sampling increases the microbiological base for interpreting syndromic surveillance. Fourth, the age stratification syndromic surveillance with a multivariate space-time can facilitate the quick interpretation of clusters by revealing the affected age groups. Next, hospital -based syndromic surveillance could be helpful in detecting local LRI-outbreaks. Finally, syndromic surveillance might be most valuable for outbreaks due to uncommon or novel pathogens like SARS outbreak.	5
Weirong Yan et al., 2013 [75]	ISSC project - An integrated surveillance system for infectious disease in rural China:	Records + Staff	10 targeted symptoms (Cough, Sore throat, Fever, Headache, Diarrhea Vomiting/nausea, Rash, Mucocutaneous hemorrhage, Convulsions, and Disturbance of consciousness)	China	Retrospective	Effective (Complement)	First, this system helps in the early detection of infectious disease epidemics as it provides near real time syndromic data collection, interactive visualization, and automated aberration detection. Second, it has the potential for identifying the change of disease patterns within the community.	6
Schenkel, K. et al., 2006 [76]	Enhanced Surveillane of Infectious Diseases	Enhanced surveillance	Accelerate and sensitise the pre-existing surveillance system for infectious diseases	Germany	Prospective	Effective	First, the delay of the notified transmitted data from the community to the federal level was reduced up to one day from three days. Second, it had the ability to detect the World Cup-related Norovirus outbreak. Third, the transformation from weekly to daily implementation was considered as a successful strategy. Next, it had the ability to intensify communication and action-oriented cooperation between players in the German public health system, In addition, this benefited the routine infectious disease surveillance in Germany.	6
White, P. et al., 2017 [77]	Mass gathering enhanced surveillance system	Enhanced surveillance	Acute fever and rash Watery diarrhoea, Non-watery diarrhoea, Influenza-like illness, Prolonged fever, Chikungunya-like illness, Dengue-like illness, Acute flaccid paralysis, Neonatal tetanus, Fever and jaundice, Acute fever and neurological symptoms Foodborne diseases	Samoa	Retrospective	Effective	First, this system is sensitive. Second, it is performed well in terms of providing vital disease early warning and health security assurance. Moreover, it had the potential of enhanced surveillance to be sustained when it is integrated from mass gathering surveillance into long-term surveillance.	4
Paul White et al., 2018 [78]	Enhanced syndromic surveillance system for mass gatherings	Enhanced surveillance	8 syndromes (Acute fever and rash, Watery diarrhoea, Non-watery diarrhea, Influenza-like illness, Prolonged fever, Fever and jaundice, Heat-related illness, Foodborne disease syndrome)	Federated States of Micronesia	Retrospective	Effective	First, this system is sensitive and specific. Second, introduction of the web-based system greatly improved the timeliness of data entry along with the analysis and SitRep dissemination, assuring the Games organizers that communicable disease would not adversely impact the Games. In addition, this has demonstrated the need for a good planning and preparation of at least 12 months to test the web-based surveillance tools, and to test methods for timely manual data collection. Finally, this system helped in understanding the importance of adequate staff resourcing to address staff fatigue due to intense daily operation of the surveillance for multiple weeks.	3
C. J. WILLIAMS,K. et al., 2009 [79]	–	Enhanced surveillance	Strengthen and augment the existing surveillance structures (SurvNet); measles, scarlet fever, chickenpox, hand foot and mouth disease, erythema infectiosum, legionellosis, Norovirus, gastroenteritis, pertussis and Salmonella.	Germany	Retrospective	Effective	First, syndromic surveillance may not be the best response to infectious disease surveillance at international events. Second, the surveillance of satisfactory events can be achieved through temporary adaptations of an existing routine infectious disease surveillance. Third, this has improved the timeliness and detection of relevant events without requiring extra sources. Finally, this has offered a chance to try out accelerated routine reporting.	6

Fifty studies reported the effectiveness of Syndromic Surveillance Systems (SSS) and the remaining 18 focused on EWSs monitoring diagnostic diseases. Forty-two articles found EWSs successfully functioned independently as surveillance systems for pandemic-wide infectious diseases outbreaks, 16 showed complementary roles thus having contributing surveillance features but cannot be relied upon solely, and 3 studies demonstrated EWS ineffectiveness. The EWS’s evaluation results of the remaining 7 articles are as follows; adjunct (*n* = 2), partial (*n* = 1), potential (*n* = 1), limited (*n* = 1), not effective but useful (*n* = 1), in addition to one effective EWS but with less capacity [66].

We summarize evidence on the effectiveness of EWS in the detection of infectious diseases outbreaks according to the source of data collection, comprised of 7 categories: emergency care and triage-based (*n* = 20), hospital/public health records (*n* = 13), web/internet-related (*n* = 11), healthcare workers-based (*n* = 13), pharmaceuticals sales (*n* = 2), and laboratory results (*n* = 1). Furthermore, EWSs involving combinations of former designs (*n* = 4) and those related to enhancing the existing traditional surveillance (*n* = 4) were classified as a separate category.

### Emergency care and triage-based EWSs (*n* = 20)

Emergency care and triage-based early warning systems gather patients’ complaints at the first contact point with the healthcare system. This EWS category includes syndromic data from Emergency departments (*n* = 11), Telephone triaging (*n* = 7), and Ambulance dispatches (*n* = 2).

#### Emergency departments - ED-EWS (*n* = 11)

All ED-EWS studies were in high-income countries (HICs) and distributed as the following; among all the studies 3 were from Australia (New South Wales NSW), [14, 16, 18] and the rest was divided into 3,2,1,1,1 for the United States [17, 19, 23], Italy [13, 21], UK [15], Taiwan [20], and Korea [22], respectively. The majority demonstrated overall good capacity as an EWS where performance evaluation revealed the independent effectiveness of 5 systems (US [17], Italy [13], UK [15], Taiwan [20], Korea [22]) and the complementary usefulness of 3 (US [19], Italy [21], Australia [18]) in addition to the supplementary effectiveness of one US EWS [23]. Only 2 Australian ED-EWSs were deemed either of mere potential benefit or ineffective overall [14, 16].

The ED-EWS competency is attributed to the ability of public health institutions to rapidly respond to cases identified by emergency departments [13, 17]. For instance, the New York City Department of Health and Mental Hygiene syndromic surveillance system (DOHMH) managed to credibly prompt public health actions related to the rise in meningitis-related ED visits, [17] and the Italian ED-SSS successfully predicted the first measles case by 2 months despite the low virus circulation documented by other traditional systems [13]. Additionally, the UK ED-SSS highlighted the privileges of hospital emergency department data in terms of being representative and inclusive to severe cases and non-residents (open access EDs), respectively [15]. This facilitates monitoring the spectrum of common pathogens, augmenting community-based surveillance besides introducing novel clinical indicators to the chief complaint - such as data on discharge status, investigations, and treatment [15].

However, the 3 studies concluded that ED effectiveness as a complement to traditional surveillance, [18, 19, 21] was due to the effects of human factors and defects in categorizing syndromes [18]. For example, Muscatello et al. pointed out that data accuracy of the Australian system was influenced by the knowledge of ED staff for medical coding and their ability to concentrate during busy shifts, especially when manifestations are documented after the visit or event or not recorded at all [18]. Particularly, the Australian information system allowed entry of only one diagnosis code per patient which might reflect a limited part of the presenting syndrome [18].

Interestingly, the only non-effective ED-SSS among the included articles was designed to only track a single symptom rather than a syndromic group of symptoms [14]. An example of this is that the passive surveillance through lab reporting of pertussis cases was 7 days more proactive than surveilling ED visits with cough [14]. On the other hand, the only included ED-EWS of adjunct effectiveness was in a recent study surveilling COVID-like symptoms rather than influenza-like illnesses [23].

The five effective ED-EWSs all noted that improving data quality is key for ED-based surveillance. Hope et al. emphasized the variation among hospitals’ data coding is an important challenge to ED-SSS [16]. Moreover, accurate diagnosis codes enabled the Korean ED-based EWS to predict influenza cases 2 weeks in advance via monitoring fever as a the chief complaint [22]. Accordingly, Joseph Wu et al. recommended the adoption of the error-detection function to enhance the efficiency and completeness of data entry, [20] given that the reliance on search-based text strings does not take into consideration misspellings, abbreviations, and synonyms [13]. Therefore, methodological approaches should target word analysis and ensure a standardized format for data extraction, and consequently, overcome the major challenge of the chief complaint syndrome surveillance model [15, 17].

Nevertheless, standardized data entry methods might have unforeseen drawbacks. Terry et al. mentioned that standardization of ED chief complaints will not be broadly applicable, given the idiosyncrasies of hospitals in entering chief complaints [19]. For instance, Westchester County’s SSS, which relies on less than three complaints to generate a signal, carries the risk of misclassification of text terms into syndrome categories which would trigger false alerts [19].

#### Telephone triaging (*n* = 7)

Only one of the seven included articles on telephone triaging-based EWS was conducted in a low-income country (Yemen) [27]. Evaluations demonstrated the effectiveness of this designated EWS category; however, the three UK National Health Service telephone helpline (NHS24) studies showed mixed results, [25, 26, 30] and the Canadian Telehealth Ontario toll-free helpline revealed a restricted role as a complement that could not be relied upon solely [24].

Assessments demonstrated telephone triaging effectiveness and strengths in terms of simplicity, health staff acceptability, and national representativeness [26, 30]. For instance, Caudle et al. declared that the lack of necessity to aggregate data from unlinked sources is an advantage for Telehealth over ED-based EWSs [24]. The Japanese telephone triaging successfully detected seasonal influenza and pediatric rotavirus outbreaks by surveillance of fever and diarrhea, respectively [28, 29].

Notably, the timeliness of the telephone triaging EWSs varies with resource availability. For example, in England and Wales, the NHS24 was capable of observing rises in syndromes within 12–36 hours from receiving calls and had successfully detected ILI outbreaks and abnormal rises in vomiting and diarrhea [26]. Additionally, Kavanagh et al. asserted the timeliness of the Scotland NHS24 in comparison to media outlets [30]. However, in low-resource settings, the timely response of phone-based surveillance systems is a major shortcoming [27]. For instance, the National Electronic Disease Early Warning System (eDEWS) in Yemen had a 2.85-day lag between the first reported case of Cholera and the initial public health response, and the duration to inform health authorities and responses’ timeliness vary from region to region [27].

Despite the above benefits, telephone triaging-based EWS has several system limitations. For example, not only is the NHS helpline likely to overlook small localized outbreaks, but also during routine surveillance the telephone calls could be a barrier to accurate data and tracking thus raising false positive alerts and triggering unnecessary public health responses [30]. Cooper et al. also revealed that the NHS helpline calls about diarrhea failed to detect a historical Cryptosporidiosis outbreak [25]. Moreover, during pandemic spread, the NHS24 alarm system becomes less sensitive, and its role becomes limited to tracking temporal changes [30]. Cooper et al. expanded that the NHS24’s full capacity will not be reached unless there is a huge surge in call rates [25]. Eventually, in the case of pediatric infectious diseases such as Rotavirus, objective reporting of symptoms by children’s parents represents a bias [28, 29].

#### Ambulance dispatches (*n* = 2)

Two studies in HICs reported that ambulance dispatches-based surveillance systems are sensitive with few false alerts [31, 32]. For instance, the independently effective US surveillance system identified the expected annual influenza epidemics in simulated serial daily analyses from 1994 to 1998 and prospectively from 1999 to 2002 [31]. While the Spanish System for Information on Detection and Analysis of Risks and Threats to Health (SIDARTHa) cannot be relied upon solely (complement) and successfully indicated the onset of high influenza activity 1 week before and at the same time as the sentinel system, in 2010–11 and 2011–12 influenza seasons, respectively [32].

Notably, surveillance systems based on ambulance service run sheets necessitate the availability of timely population-wide electronic data that are routinely collected and liable to categorization into syndromes [31, 32]. Without real-time data, the designated systems would reach an undesirable sensitivity where the missing number of influenza isolates for a particular day will not be associated with a rise in the alarm threshold of that day [31]. Moreover, the Spanish system for Information on Detection and Analysis of Risks and Threats to Health (SIDARTHa) was able to utilize the call logs and ED patient records at the same time from the medical dispatch center [32]. This shows the complementary action and the potential of use for more than one EWS [32].

### Hospital/public health records (*n* = 13)

Hospitals and public health registries are indispensable for monitoring infectious diseases. The majority of the included EWSs in this category proved independent effectiveness (*n* = 10) apart from two complementary and one partially effective system in LICs.

#### Hospitals and health facilities records (*n* = 7)

Seven of the studies evaluated hospitals and health facilities-related EWSs covering electronic or paper-based results of the hospital admissions (*n* = 1) and the inpatient data (*n* = 6).

Admission-based EWSs are preferable to be implemented in states with “discrete population centers” and run by staff “aware of hospital admission patterns” to increase the likelihood of unusual event recognition [35]. Although admission-based systems do not capture outpatient illness-related outbreaks, they are advantageous in identifying and provoking investigations for unusual syndromes and those limited to one case [35]. For instance, the US Hospital Admissions Syndromic Surveillance statewide syndromic surveillance (HASS) proactively recognized the rare condition of West Nile virus encephalitis and one-case outbreaks such as smallpox or SARS [35]. Nevertheless, Dembek et al. stated that the inpatient SSS has an average lag of 1–2 days in comparison to the outpatient one given the time lapse from manifestations onset and admission [35]. Additionally, reliance on case counts overlooks potentially useful demographic data [35].

The articles on inpatient EWSs adopted either an ICD-diagnosis code-based system or Symptom-Clicking-Module (SCM) for automatic grouping of symptoms through “pre-defined syndrome definitions” [33, 34, 38].

Although inpatient EWSs investigate data on patients with different risk factors and conditions, a German study demonstrated the potential system’s in-season ineffectiveness since cases with comorbidities tend to have longer hospital stays with delayed inpatient data collection, in addition to the absence of laboratory confirmation results [34]. However, inpatient systems could permit manual exploration of time series figures of hospitals’ daily surveillance data by local epidemiologists [33]. Moreover, Ang. et al., evaluating the ICD-9-based Singaporean Patient Care Enhancement System (PACES), recommended that “repeat consults” would abolish the inherent background noise of the primary care consult-based system and help detect outbreaks of sizes undetectable by community-based systems [38].

In resource-limited areas, such case definition-based EWSs are associated with implementation challenges. For example, the challenging contextual situation in Yemen impedes timely reporting and alert verifications where “only 21% of all the National Electronic Disease Early Warning System (eDEWS)‘s alerts were verified within the first 24 h of detection in 2016” [36]. Accordingly, the local health system fragility along with poor “understanding of case definitions” contributed to having quality and timeliness as major eDEWS’ drawbacks [36]. Also, the Indian indicator-based surveillance (IBS) and event-based surveillance (EBS) systems encounter challenges in terms of gathering electronically-delivered data from reporting units, absence of previous baseline data, and incomplete indicator-based surveillance data capturing (admitted patients in the central hospital) [37]. Furthermore, the tendency for data-burden reduction in deprived settings is another hurdle in LMICs. For instance, the simplicity of the Pacific island countries and territories (PICTs) through reliance on a definite number of easily assessed syndromes entailed that cases will not be notified unless they meet the included case definitions and that a rise in symptoms will not generate alerts [39].

#### Public health records (*n* = 6)

Public health (PH) records from public health offices reflect epidemiological data ranging from the incidence of different notifiable infectious diseases to all-cause mortalities. PH records-based EWSs were assessed in six studies; among the total number of studies four were in LMICs [41–44] and two in HICs [40, 45]. All demonstrated independent effectiveness except the Ghana PH system of partial competency [42].

Public health records-based EWSs successfully rely on centralized systems facilitating information sharing across local public health departments [41, 43, 44]. For instance, the Chinese Infectious Disease Automated-alert and Response System (CIDARS) collects data from the existing electronic National Notifiable Infectious Diseases Reporting Information System (NIDRIS) and reports to CDCs via short message service (SMS) [43]. Zhang et al. asserted CIDARS’s high sensitivity and specificity with a 3-day median detection time has shown effectiveness in detecting Dengue Fever (DF) outbreaks [44]. Moreover, a prototype EWS in Brazil tracking epidemiological viral circulation data during the 2014 World Cup had effectively “predicted high risk of dengue for 57% of the microregions reporting high levels of dengue during the games” and provided warning 3 months in advance [41]. Also, through real-time data analysis from existing Provincial Electronic Health Record data, the Alberta Real Time Syndromic Surveillance Net (ARTSSN) in Canada overcame the traditional system fragmentation, taking the advantage of “timeliness, comprehensiveness, and automation [40]. Furthermore, Ghana PH-EWS utilizes the National Influenza Center (NIC) routine ILI data where it detected circulating influenza A and B lineages with an “average of 10 days between symptom onset and detection“ [42]. Eventually, the French death certificates-based EWS is an “all-cause mortality surveillance system” compiling data “from computerized city halls” [45].

However, in PH records-based EWSs, outbreaks would not be detected until clinicians reported cases [43]. Yang et al. demonstrated CIDARS’s potential less timeliness and sensitivity in comparison to EWS based “on data on pre-diagnosis of cases in hospitals, media reports or school absenteeism” [43]. In addition to the previous routinely accepted delays, a French study by Baghdadi et al. showed that there is an additional hurdling delay (90% of mortality within 7 days) in the web-based death certification by physicians in the French system “Reactive mortality surveillance system- syndromic surveillance system SurSaUD” [45].

### Web and internet-based EWSs (*n* = 11)

#### Web-based early warning systems (*n* = 4)

Four studies evaluated Web-based EWS using computer software; three studies in LMICs (China [47], the Republic of Macedonia [48], India [49]) and a single one in HICs (Norway [46]). The designated systems monitored an array of syndromic groups except China’s infectious disease automated alert and response system which focused on Hand, foot, and mouth disease (HFM) [47]. Assessments demonstrated independently the effectiveness of Web-based EWSs except for the complementary role of ALERT in the Republic of Macedonia [46–48].

Web-based Early Warning Systems enhance communication across surveillance networks from health facilities at local levels to higher public health authorities [46, 48]. For example, Li et al., evaluating the Chinese web-based alert and response system, revealed its sensitivity especially with larger outbreaks (> 20 cases) than smaller ones (< 10 cases) with an average detection time of 1.7 and 2.1 days, respectively, and a 4.5 days-lag until reporting to public health authorities [47]. Additionally, a “digital disease surveillance system”, relying on cell phone applications, allowed the Indian health authorities to monitor mass gathering surveillance data through “near-real-time daily reports” from local health staff and epidemiologists [49].

Notably, facilitated reporting by remote health settings and rapid alarm transmission are key advantages of Web-based EWSs. For instance, the web-based ALERT system of Macedonia, relying on primary care facilities’ data, had successfully detected the onset of seasonal influenza and was more proactive than the routine diagnosis-based surveillance [48]. Moreover, Guzman-Herrador et al. revealed that in the Norway web-based system, Vesuv, efficient information exchange allowed the update of outbreak data, and had easy log-in by various stakeholders, besides sending automatic reminders to notifiers within 3 weeks [46].

#### Internet-based early warning systems (*n* = 7)

Seven studies evaluated internet-based systems; Email-based systems (*n* = 2) in Darfur [50] and Netherlands [51], and Social trends-related EWSs (*n* = 3) in the US [54], Europe [56], and Canada [55] which revealed independent effectiveness. In addition, 2 studies reported contradicting results regarding the effectiveness of the US Electronic Surveillance System for the Early Notification of Community-based Epidemics (ESSENCE) [52, 53]. A study additional to the previous seven studies- was categorized under pharmaceutical-based EWS category- was published by Dong et al. stated that the Chinese Baidu search queries–for the “fever” term–showed a strong correlation with influenza activity, and along with Sentinel hospital ILI and over the counter (OTC) drug sales could complement the routine surveillance based on lab-confirmed cases [70].

The covered email-based systems –which rely on emails as a means of communication between the EWS staff– compile surveillance data from various resources; media and official reports, online sources, and local observers, which are later transmitted to national and federal levels [50]. Studies revealed that email-based systems have unique considerations regarding data quality and the need for key informants. For instance, the email system in Darfur’s refugee camps demonstrated the inherent risk of inaccurate data documentation and imprecise information about the camps’ population [50]. Moreover, Zeldenrust et al., evaluating the Netherlands ProMED-mail (Program for Monitoring Emerging Diseases), stated that the system specificity is low as its information sources are liable to bias and some lack scientific terminologies [51].

Likewise, studies on social trends-related EWSs –tracing infectious diseases symptoms and signs shared by users of social platforms– raised concerns over unethical and biased data collection [54–56]. For instance, data storage and utilization of Google Flu Trends is done without consent, jeopardizing users’ privacy issues [54]. Samaras et al. emphasized that internet data cannot be 100% accurate which is subjected to underestimate or exaggeration depending on human reaction to situations [56]. Furthermore, educational level along with cultural and language backgrounds affect the accuracy of symptoms shared by internet users [54]. For instance, Dong et al. stated that the Chinese Baidu search queries for the “fever” term are affected by health-seeking behavior such as fear or curiosity that would affect the public search queries [70]. Nevertheless, according to Betancourt et al., the US Electronic Surveillance System for the Early Notification of Community-Based Epidemics (ESSENCE)–an internet-based system utilizing ICD-9–is effective given its data completeness, accuracy, and high specificity (95.5–96%) [53].

Moreover, representativeness is a huge hurdle to social trends-based EWSs. For example, data sampling and computational approximation methods influence the data accuracy of Google Flu Trends [54]. Dion et al., evaluating the Canadian Global Public Health Intelligence Network (GPHIN), also asserted the representativeness limitation of social trends-related EWSs since social media platforms are not accessible to everyone, as well as social tweets’ word limit might prevent the inclusion of potentially useful contextual information, respectively [55].

Despite the above-mentioned concerns, monitoring social trends has successfully functioned as an early warning system in HICs with speedy data processing as a key advantage. For instance, the real-time operation on a 24/7 basis gave GPHIN a remarkable velocity where data retrieval and processing occurred every 15 and less than 1 minute, respectively [55]. The US Google Flu Trends was 1–2 weeks more proactive than CDC reports in estimating levels of influenza [54]. Also, the European Google Flu Trends in France, Germany, the UK, and Spain proactively detected measles outbreaks within 2 months [56]. However, as for Witkop et al., ESSENCE has a low positive predictive value (31.8%), triggering “time-consuming false alarms”, and failing to detect influenza outbreaks early enough with a time lag of 1–3 days [52].

### Human resources-based (*n* = 13)

Thirteen articles evaluated a unique category of early warning system that relies on human resources to proactively detect outbreaks. The involved cadres range from general practitioners (*n* = 5) [58, 59, 62, 67], community health workers (*n* = 2) [60, 63], volunteers (*n* = 3) [57, 61] to school staff (*n* = 3) [64–66]. Unlike other EWS categories, human resources-based systems are implemented more in LMICs (Ghana [60], Sierra Leone [63], Madagascar [62], Iraq [68] and China [66]), and even in one of the HICs (Austria) [57] the system monitors outbreaks among former residents of developing countries. None of the ESWs in this category were ineffective; whereas, effectiveness varied from independent functioning (*n* = 6) [12, 57, 59–61, 63] to complementary effectiveness (*n* = 6) [58, 62, 64, 65, 67, 68], in addition to one showing less capacity than the artificial intelligence-based form [66].

#### General practitioner (*n* = 5)

GPs participation in EWSs ranged from voluntary participation from different provinces or recruited from defined clinics, to Out-of-Hours services and house calls network [58, 59, 62, 67]. The included studies were implemented in HICs (France [58], New Zealand [59], and the Netherlands [67]) except the one in Madagascar [62] and that in Iraq [68]. All had complementary functions (*n* = 4) except the independently effective one in New Zealand (*n* = 1) [59]. GP-based EWSs surveil a broader range of symptoms in comparison to other EWS categories [58, 59, 62, 67, 68]. The reason is that man-powered surveillance systems offer resilience and flexibility with data acquisition [58, 59, 62, 67].

Assessments of the general practitioner-based EWS underscored the real-time nature of this category of surveillance systems which has several pros both from the user and public health perspectives. From the physicians’ perspective, Flamand et al. stated acceptability of the French SOS Medecins by GPs as their role was limited to routine data gathering, without additional work requirements [58]. Furthermore, an evaluation of the Netherlands ICARES (Integrated Crisis Alert and Response System) asserted that the system does not impose a work burden [67]. Jones N. F. and Marshall R., evaluating the New Zealand EWS, reported the increasing compliance of the participating GPs [59]. Eventually, compliance of the participating Madagascar system’s GPs was evidenced by the 89% estimated rate of daily data transfer [62].

Regarding the public health perspective, the Netherlands ICARES generated alerts within 24 hours from data entry by the treating practitioner [67]. Also, Randriana et al. mentioned that the Madagascar EWS successfully detected rises in fever manifestations related to influenza, arbovirosis, and malaria [62]. Furthermore, deploying SSS assisted Iraqi health staff to recognize rises in influenza-like illness by combining surveillance data on “fever and cough symptoms” during mass gathering events [68]. Eventually, the French EWS showed high sensitivity and being human-operated, offering flexibility to changes in syndromes under surveillance, along with access to illness data that are outside of hospital admissions [58].

Additionally, human factors-related considerations might impede the GP-based EWSs’ full capacity. Firstly, the Madagascar Sentinel syndromic-based surveillance system highlighted that GP-based EWSs are likely to be associated with relative tardiness in eliciting public health responses, which might outweigh the advantage of rapid data gathering [62]. Accordingly, Jones N. F. and Marshall R. pointed out the importance of proper planning of resource allocation in terms of GP time to ensure system maintenance [59]. Nevertheless, the Madagascar experience was encouraging for developing countries; however, ensuring robust communication systems with competent internet connections is the main challenge [62]. Secondly, bias arising from GPs’ recording behavior is another human factors-related hurdle for GP-based EWSs.

#### Community health workers (*n* = 2)

Community-based surveillance in the included studies encompasses recruited community health monitors and surveillance supervisors where reporting followed a bottom-up approach from districts to public health authorities [60, 63]. Two studies demonstrated the independent effectiveness of community-based surveillance (CBS) in LMICs [60, 63]. For instance, the modified CBS in Ghana detected over 300 events that would otherwise go undetected [60]. Also, the new Ghana EWS identified 26% of all suspected vaccine-preventable disease cases reported by routine surveillance [60]. Additionally, the Community Event-Based Surveillance (CEBS) in Sierra Leone demonstrated rapid cases detection in comparison to the national system and it had successfully triggered alarms to one-third of Ebola cases identified [63].

The authors highlighted obstacles that are unique to Community-based Surveillance. The CBS broader-scale implementation is challenging given the required extensive training for community members and maintaining communication with the surveillance team [63]. Moreover, like the GP-based EWSs, Community-based Surveillance is liable to biases from staff miscategorization that would lead to inaccurate capturing of cases [63]. Furthermore, monitoring of death events (late indicator) by community health workers could be challenged by the practice of unsafe burials such as in Sierra Leone CEBS [63]. Accordingly, Ratnayake et al. recommended boosting community engagement, adjusting trigger definitions, and full involvement in the overall Integrated Disease Surveillance and Response system [63].

#### Others (*n* = 6)

Reliance on volunteers and school staff requires additional involvement of human resources for the early detection of infectious disease outbreaks. Three effective manpower-based EWSs recruited focal persons at asylum seekers’ reservation centers, and volunteers at Hurricane Katrina and Hurricane Harvey evacuees’ shelters in Austria and the US, respectively [12, 57, 61]. For instance, an acute gastroenteritis outbreak and tracking a rise in respiratory symptoms were successfully confirmed by the American volunteers-based EWS [61]. Lastly, two complementary effective School-Based Syndromic Surveillance System (SID-SSS) in Taiwan and UK gathered surveillance data “from school nurses or class teachers (for those preschools without nurses)” and school absenteeism reports, respectively [64, 65]. However, Yang et al. highlighted that utilization of information technology to record school absenteeism has better “simplicity, cost-effectiveness, data quality, sensitivity, and timeliness” metrics than manual approaches by school staff (completeness of 100% versus 86.7%, respectively) [66].

This EWS category surveilled an array of infectious diseases’ prodromal manifestations that are likely to develop in crowded settings [12, 57, 61, 64]. Gathering pre-defined data sets from confined populations allowed near real-time data collection with timely public health response [12, 57, 61]. For instance, the US Emerging Disease Syndromic Surveillance’s cot surveys took less than 60 seconds (to 5.2 minutes) per person, which enabled daily assessment of evacuees [12, 61]. Also, El-Khatib et al. pointed out the practicability of tally sheets of the Syndrome-Based Surveillance System (SbSS) for Infectious Diseases among Asylum Seekers which monitor easily recognizable manifestations of infectious diseases [57]. The Austrian system is a reliable one with high sensitivity and had the advantage of rapid implementation in emergencies [57]. Moreover, surveilling school children in Taiwan–for students’ absenteeism and family health status besides clinical and epidemiological data–addressed gaps within ED-SSS since the former tracked mild cases at pre-diagnosis levels while the latter collected data on severe illnesses from all age groups throughout the year [64].

Nevertheless, low specificity and false-negative reports are potential drawbacks of the designated EWS category [61]. For example, the SbSS for Infectious Diseases among Asylum Seekers did not have consistent availability of staff at reservation centers nor accurate daily registration of refugees, and if asylum seekers were admitted to hospitals immediately, underreporting and under-detection of syndromes are potential challenges [57]. Additionally, Murray et al. disclosed that evacuees – to avert isolation placement – were likely to hide illnesses [61]. Moreover, Weng et al. revealed that school breaks and reporting variations are common limitations for SID-SSS [64]. Although the school-based influenza-like illness (ILI) surveillance in China was 1 week more proactive than lab confirmation, Dong et al. asserted that inability to track relevant data during holidays and weekends leads to inaccurate assessment of “community-level influenza activity during these periods” [70]. Eventually, specificity of school-based syndromic surveillance system could be enhanced by including virological surveillance of representative samples [64].

### Pharmaceuticals sales (*n* = 2) and laboratory results (*n* = 1)

Monitoring over-the-counter (OTC) medications sales and diagnostic laboratory-based testing represent surveillance means where early warning systems trace patients’ data from paramedical sources. None of the 3 EWSs included in the designated category proved their independent functionality [69–71].

Laboratories compliance and clinical correlation are the main challenges to lab-based EWSs. For example, the Netherlands’ Infectious diseases Surveillance Information System (ISIS) had low coverage where only 18 out of the 85 included labs were connected to the central medical microbiology laboratories (MML) [71]. Thus, the collected positive and negative microbiological results were not routinely gathered into the laboratory information management systems in a representative manner for the Netherlands [71]. Moreover, laboratory influenza specimens do not reflect the total viral activity [70]. For instance, respiratory pathogens such as a respiratory syncytial virus (RSV) have similar manifestations to influenza, thus would weakly correlate with lab ILI positive results [70]. Nevertheless, discontinuation of the above ineffective systems is not recommended where lab EWSs provide up-to-date trends of micro-organisms incidence and their suspension would lead to loss of important epidemiological data [71].

On the other hand, the inability to trace back cases represents a major pitfall in relying on drug prescription sales for surveillance. For example, the unavailability of information of the OTC purchasers limited the effectiveness of the drug sales EWS in Tianjin as a complement to existing influenza lab surveillance [69]. Nevertheless, despite the New York City OTC-EWS data collection on prescription sales and information on medical visits besides OTC drugs, Das et al.’s also mentioned that the designated New York system acted as an adjunct for surveillance where gastrointestinal drug sales were less sensitive than ED diarrheal visits where its effectiveness is limited to monitoring patients’ level [69].

Additionally, consumer behavior and the evolving OTC market present unique challenges to pharmaceutical sales-EWSs. For instance, if the public is stockpiling OTC medicines, this would mask the real consumption from acute illness [69]. Moreover, new medications’ market entry and the variety of drug formulations represent challenges for categorizing syndromes of the OTC sales-based EWS [69].

### Multi-data source (*n* = 4)

The multi-source EWSs in the included articles rely essentially on data records– for syndromic surveillance of infectious disease outbreaks–either by combining different kinds of health registries or those with various data-gathering methodologies. Three of these EWSs were conducted in the Netherlands; two retrospectively revealed successful combined effectiveness, and one showed a complementary role by Predictive simulation [72–74]. The fourth study demonstrated the complement effect of the Chinese multi-source EWS by retrospective assessment [75].

Leveraging Records and Staff was one of the successful EWS combined designs. For example, Wijngaard et al. reported that the Netherlands EWS used a combined design, hospital data from the National Medical Register, and ILI data from a sentinel network of GPs, facilitating a timely detection of localized outbreaks - especially those related to emerging pathogens, independently of lab surveillance [74]. Wijngaard et al. also pointed out that detection proactivity of different data sources–especially for respiratory infectious diseases surveillance–is as follows; “hospital data (+1 week), pharmacy purchases/GP consultations (+2 weeks), and deaths/lab diagnostic requests (+3 weeks)” [73]. However, the latter associations should be further validated, especially those related to “absenteeism and pharmacy data” [73]. Additionally, rural China (ISSC project); utilized manual labor from health facilities, pharmacies, and primary schools for data entry from paper forms into automated electronic data capture, enabling timely identification of epidemics with recognition of changing disease patterns [75].

Combining public health reports and email services is another successful multi-data source EWSs. In the Netherlands, monitoring the daily ECDC Round Table Report and ProMED-mail was associated with the timely reporting of 95% of threats [72]. Bijkerk et al. also highlighted that this combined EWS approach would “save at least 2.5 hours a week on human resources” [72].

### Enhanced surveillance (*n* = 4)

The four included articles evaluating Enhanced Surveillance Systems revealed the effectiveness of their temporary adaptations for mass gathering events [76–79]. The systems’ enhancements targeted the frequency of epidemiological data transmission with 2 studies agreeing that the daily surveillance data notification was the most successful and accepted enhancement strategy [76, 79]. For instance, the Germany-based Enhanced Surveillance Systems during the 2006 FIFA World Cup had adaptations in terms of days instead of weekly transmission of notifiable diseases, marking World Cup-related cases in SurvNet – the routine surveillance system, additional daily reports from the hosting cities besides the daily analysis and reporting by the National Enhanced Surveillance Operations Centre (NESOC) [79]. Williams et al. confirmed that the latter modifications had strengthened the timeliness and detection capacity without the substantial need of additional resources and that it is unnecessary to implement syndromic surveillance in mass events unless the routine reporting system is not robust [79].

Another form of temporary enhancements, World Cup German Enhanced System, included accelerating data transmission to the existing electronic surveillance system through immediate telephone contacts, allowing free-text notifications even if outside the included case definitions, media monitoring for relevant events, and boosting communication between national and international stakeholders [76]. Schenkel et al. highlighted that the latter system adaptation sensitized the routine mandatory notification system where it has successfully detected a Norovirus outbreak related to the World Cup [76]. Additionally, 1 day was the average data transmission time to federal authorities, instead of three [76].

In LMICs, evaluating the benefits of enhancing existing systems over syndromic surveillance is controversial, given that sustaining daily data transmission is challenging in low-resource settings [76]. For instance, full utilization of a web-based tool of Suite for Automated Global Electronic bioSurveillance Open ESSENCE (SAGES OE) for data storage in the Federated States of Micronesia and Samoa, within the context of the 8th Micronesian Games and the third United Nations Conference on Small Island Developing States (SIDS), retrospectively, was challenged by connectivity issues and lack of enough computers and trained staff [77, 78]. Furthermore, a spreadsheet-based alternative was used in SIDS due to SAGES OE technical limitations [77]. Nevertheless, authors highlighted that the designated enhanced surveillance helped in decision-making and acted as an assurance system for health security with the potential of full integration into routine surveillance as it boosted communication channels between clinical, laboratory, and public health departments [77, 78].

## Discussion

To our knowledge this review is the first to synthesize evidence on the evaluated effectiveness of Early Warning Systems (EWSs) for proactive detection of infectious diseases outbreaks. The majority of the studies were in HICs and those included from low and middle-income countries were almost equal. Most of the studies evaluated Syndromic Surveillance Systems (SSS) and a few focused on EWSs monitoring diagnostic diseases, assessing systems with an array of methods for surveillance data collection. Emergency departments (ED) and triaging data in addition to those from hospitals and public health records formed the main bulk of data sources for the included EWSs. Furthermore, data from the following surveillance methods were included: web/internet- and healthcare workers-based EWSs. Additionally, a few studies covered pharmaceutical sales, laboratory results, multi-design EWSs, and enhanced traditional surveillance systems.

There is consistent evidence that Inpatient SSS has an average lag of 1–2 days in comparison to the outpatient one, given the time lapse from manifestations onset and admission [35]. Moreover, in Singapore Ang. et al. assessment demonstrated that the retrospective study was not able to evaluate the staff sick leave’s impact on surveillance systems relying on PACES (in-patient) [38].

Most studies that included Public Health records-based EWSs highlighted that outbreaks would not be detected until clinicians reported cases [43]. For instance, Yang et al. demonstrated (PH-EWS) CIDARS’s potential less timeliness and sensitivity in comparison to EWS based “on data on pre-diagnosis of cases in hospitals, media reports or school absenteeism” [43]. Moreover, Kavanagh et al. asserted the timeliness of the Scotland NHS24 (telephone triaging) in comparison to media outlets [30].

Studies on General Practitioner-EWS underscored the real-time nature of this category. The few studies that looked at school surveillance pointed out that students’ absenteeism and family health status, besides clinical and epidemiological data, addressed gaps within ED-SSS since the former tracked mild cases at pre-diagnosis levels while the latter collected data on severe illnesses from all age groups throughout the year [64]. There was also evidence that school surveillance was 1 week more proactive than lab confirmation [70]. However, for example, passive surveillance through lab reporting of pertussis cases was 7 days more proactive than surveilling ED visits with cough [14]. Additionally, Das et al. mentioned that the US OTC-EWS merely acts as an adjunct for surveillance; for instance, gastrointestinal drug sales were less sensitive than ED diarrheal visits [69].

With multi-source EWSs, Wijngaard et al. stated that detection proactivity of different data sources–especially for respiratory infectious diseases surveillance–is as follows; “hospital data (+1 week), pharmacy purchases/GP consultations (+2 weeks), and deaths/lab diagnostic requests (+3 weeks)” [73]. However, the latter associations should be further validated, especially those related to “absenteeism and pharmacy data” [73]. The few studies on death-based EWSs agreed that delayed reporting is a major drawback, such as in the web-based death certification by physicians in the French (90% of mortality within 7 days) [45]. Also, web-based EWS primary care facilities are not the optimum notification source [48]. It is noteworthy that in LMICs, evaluating the benefits of enhancing existing systems over syndromic surveillance is controversial, given that sustaining daily data transmission is challenging in low-resource settings [76]. For example, Sentinel hospital ILI, OTC drug sales, Baidu search query, and school-based ILI surveillance are a complement to traditional surveillance systems in China [70].

There is also consistent evidence that the competency of ED-EWSs is attributed to the ability of public health to rapidly respond to cases, besides ED’s data representative and inclusion of severe cases and non-residents [15]. Any recognized pitfalls were related to human factors and defects in categorizing syndromes. To sum up, using emergency department data on chief complaints and presenting symptoms is an effective EWS [19]. Nevertheless, it requires staff training on medical coding with wise utilization of standardized formats among hospitals’ EDs [18].

On the other hand, the effectiveness and strengths of telephone triaging systems were in terms of simplicity, health staff acceptability, and national representativeness [26, 30]. Notably, the timeliness of the telephone triaging EWSs varies with resource availability. However, there was evidence that telephone triaging-EWSs has several system limitations such as overlooking small localized outbreaks and less sensitivity during pandemics, besides objective reporting [28, 29].

Ultimately, evidence has demonstrated the sensitivity of ambulance dispatches EWSs with few false alerts [31, 32]. Recommendations necessitated the availability of timely population-wide electronic data that are routinely collected and liable to categorization into syndromes as crucial for ambulance dispatches EWSs [31, 32].

Evidence has revealed that admission-based EWSs are preferable to be implemented in states with “discrete population centers” and run by staff “aware of hospital admission patterns.” [35] Although they do not capture outpatient illness-related outbreaks, they are advantageous in identifying and provoking investigations for unusual syndromes and those limited to one case [35]. On the other hand, the evidence demonstrated that inpatient EWSs–relying on an ICD-diagnosis code-based system or Symptom-Clicking-Module (SCM) for automatic grouping of symptoms through “pre-defined syndrome definitions”–had in-season ineffectiveness [33, 34, 38]. However, inpatient systems could permit manual exploration of time series figures of hospitals’ daily surveillance data by local epidemiologists, with recommendations that “repeat consults” would abolish the inherent background noise of the primary care consult-based system [38]. Nevertheless, in resource-limited areas, such case definition-based EWSs are associated with implementation challenges.

The key advantage of Public Health records-based EWSs is successfully relying on centralized systems, facilitating information sharing across local public health departments [41, 43, 44]. However, in this case, outbreaks would not be detected until clinicians reported on their cases [43].

Web and internet-based Early Warning Systems carry huge potential for outbreak detection. Web-based EWSs enhance communication across surveillance networks from health facilities at local levels to higher public health authorities [46, 48]. Evidence revealed that facilitated reporting by remote health settings and rapid alarm transmission are key advantages of Web-based EWSs. On the other hand, internet-based EWSs include Emails and Social trends-related systems. Email-based systems compile surveillance data from various resources: media and official reports, online sources, and local observers that are later transmitted to national and federal levels. However, they have unique considerations regarding data quality and the need for key informants. Social trends-related EWSs raised concerns over representativeness and unethical biased data collection [54–56]. Despite the above-mentioned concerns, monitoring social trends has successfully functioned as an early warning system in HICs with speedy data processing as a key advantage.

Our analysis demonstrated that human resources-based systems are implemented more in LMICs where none of the included EWSs in this category were ineffective. The included cadres ranging from general practitioners, community health workers, and volunteers, to school nurses. First, general practitioners ranged from voluntary participation from different provinces or recruited from defined clinics to Out-of-Hours services and house calls network. The designated system monitored a broader range of symptoms in comparison to other EWS categories given that man-powered surveillance systems offer resilience and flexibility with data acquisition [58, 59, 62, 67]. Assessments of the general practitioner-based EWS underscored the real-time nature of this category of surveillance systems, which has several pros both from user and public health perspectives. However, human factors-related considerations might impede the GP-based EWSs’ full capacity, relative tardiness in eliciting public health responses, and biases arising from GPs’ recording behavior.

The community health workers’ (CHWs) include recruited community health monitors and surveillance supervisors. Obstacles of relying on CHWs encompass extensive training for community members, maintaining communication with the surveillance team, and staff syndromes’ miscategorization [63]. On the other hand, reliance on volunteers and school staff enables gathering pre-defined data sets from the confined population, and surveilling an array of infectious diseases’ prodromal manifestations that are likely to develop in crowded settings [57, 61, 64]. However, low specificity and false-negative reports are potential drawbacks [61].

The included systems for pharmaceutical sales and laboratory results did not prove solo effectiveness. Laboratories’ compliance and clinical correlation are the main challenges to lab-based EWSs; laboratories’ low coverage and results may not reflect the total viral activity [70]. Nevertheless, discontinuation of the above ineffective systems is not recommended [71]. Furthermore, the inability to trace back cases represents a major pitfall of relying on drug prescription sales for surveillance. Additionally, consumer behavior and evolving OTC market embody unique challenges to pharmaceutical sales-EWSs [69].

Evidence has also revealed that multi-data source EWSs and enhancing traditional systems are promising for early outbreak recognition. Leveraging Records and Staff was one of the successful EWS combined designs. Also, enhanced surveillance temporary adaptations showed effectiveness during mass gathering events. The systems’ enhancements targeted the frequency of epidemiological data transmission with two studies agreeing that the daily surveillance data notification was the most successful and accepted enhancement strategy [76, 79]. However, in LMICs, evaluating the benefits of enhancing existing systems over syndromic surveillance is controversial, given that sustaining daily data transmission is challenging in low-resource settings [76]. Authors highlighted that the designated enhanced surveillance helped in decision-making and acted as an assurance system for health security with the potential of full integration into routine surveillance [77, 78].

Based on our extensive analysis of both syndromic and diagnosis-based EWSs, we suggest the following combinations as appropriate enhancement strategies for infectious diseases surveillance. In HICs, we recommend that investment be directed towards the spectrum of syndromic surveillance systems –tracking pre-diagnosis data- ranging from social trends, emergency care, and triage-based EWSs, to hospitals and health facilities records. This is attributable to the abundance of facilitations and financial resources in HICs that would enable the adoption of standardized speedy monitoring of human health data. On the contrary, LMICs -given their manpower availability- are advised to allocate investment towards the existing diagnosis-based EWSs such as centralized Public Health records, web, and email-related surveillance systems in addition to human resources-based EWSs.

There is scope to explore the One-health approach, including environmental and veterinary surveillance systems besides human-based ones. Our recommendation is aligned with a recent systematic review on EWSs for “vector-borne diseases” where Baharom et al. highlighted that incorporating meteorological and environmental surveillance systems with other “epidemiological tools” is a very promising strategy for outbreaks detection [80].

Also, the fact that Syndromic Surveillance Systems (SSS) are more proactive than diagnostic disease surveillance should not be taken as an effective indicator/clue for outbreaks detection. Given that in literature, LMICs and their vulnerable highly populated areas were under-represented in comparison to high-income ones, there is a need to investigate more the implementation contextual feasibility of different EWSs’ categories in low resource settings. For instance, studies revealed that in deprived areas staff training is integral for the implementation of electronic automated surveillance.

A key strength of our study lies in the fact that we captured all human-based EWSs of pandemic scope regardless of the surveilled symptoms or ailments. Additionally, by including both SSS and diagnostic disease surveillance systems, we were able to synthesize evidence on the level of effectiveness of EWSs’ source of data collection considering the broad spectrum of mild and severe clinical presentations. However, despite it being broad in scope, the review still had some weaknesses and limitations.

First, regarding the quality of the included articles, a few studies did not have specific aims; their focus was on making general observations of the efficacy of an intervention. For instance, some did not detect outbreaks but authors reported it to be effective. Also, some of the studies included did not make comparisons with the existing mode of surveillance.

Second, we applied language restrictions to English. However, we have quite well EWSs assessed from all over the world apart from areas that usually do not implement EWSs nor publish relevant credible sources.

Third, we did not include gray literature or pre-print publications. Nevertheless, the 61 included articles covered the broad spectrum of EWSs methods we aimed for which fitted the scope of our study in terms of effectiveness for detecting potential pandemic-wide outbreaks.

Fourth, we limited our search to PubMed and Scopus databases. However, the databases of choice are comprehensive where most of the well-recognized articles are indexed. Furthermore, the yielded results from PubMed and Scopus were reassuring to be a representative sample for the available literature, as each EWS category was analyzed in a considerable number of studies, and nearly a consensus was found per each category of studies in terms of pros, cons and the recommended course of action for improvement. Nevertheless, we recommend the inclusion of additional databases for further research.

Fifth, we did not include studies focusing only on facility settings outbreaks or outbreaks on a small scale. This might have added insights into potential unforeseen warning systems for proactive public health interventions. However, we were reassured that our search strategy did not restrict the inclusion of infectious diseases under surveillance, reflecting the different data collection methodologies that were captured.

Finally, the operationalization of evaluating EWS effectiveness varied across the studies. For example, the surveillance coverage of some studies was on pre-defined groups such as refugees and schools. Although the majority were of wide scale, population-based, comments on EWSs’ specificity, sensitivity, completeness as well as subjectivity, and transparency of reporting were not included in all studies. Moreover, monitored symptoms, case definitions, and EWS alert thresholds varied. This heterogeneity in assessment parameters makes meaningful comparison difficult.

The EWSs’ assessment results are plausible with rational justifications of effectiveness for each data collection method. However, as most of the current outbreaks with potential to become pandemics are due to zoonotic diseases, there is a need for more studies on the One-health approach, including environmental and veterinary systems with human data. Such studies could explore more proactive interdisciplinary public health strategies, particularly for respiratory transmitted diseases.

Only a few studies reported on enhancing surveillance of traditional systems that were all in mass gathering events. Therefore, making conclusions from our findings should be done with caution, particularly within the context of all-year surveillance systems. There is therefore the need for exploring the potential of enhancing surveillance systems throughout the year and specifically in LMICs.

## Conclusion

Our study was able to evaluate the effectiveness of Early Warning Systems (EWSs) in different contexts and resource settings based on the EWSs’ method of data collection. There is consistent evidence that EWSs compiling pre-diagnosis data are more proactive to detect outbreaks. Emergency departments (EDs) data on chief complaints is an effective EWS but it requires utilization of standardized formats among hospitals’ EDs. Simplicity, health staff acceptability, and national representativeness are strengths of telephone triaging systems. Eventually, inpatient systems could permit manual exploration of hospitals’ daily surveillance data by local epidemiologists.

Centralized Public Health records-based EWSs facilitate information sharing; however, they rely on clinicians’ reporting of cases. Interestingly, human resources-based systems are implemented more in LMICs where none of the included EWSs in this category were reported ineffective.

Facilitated reporting by remote health settings and rapid alarm transmission are key advantages of Web-based EWSs. Email-based systems have unique considerations regarding data quality and the need for key informants. And, social trends-related EWSs successfully functioned but raised concerns over representativeness and unethical biased data collection.

Notably, pharmaceutical sales and laboratory results did not prove solo effectiveness. The EWS design combining surveillance data from both health records and staff was very successful. And, daily surveillance data notification was the most successful and accepted enhancement strategy especially during mass gathering events.

The fact that Syndromic Surveillance Systems (SSS) are more proactive than diagnostic disease surveillance should not be taken as an effective clue for outbreaks detection. Although HICs are recommended to focus investment on EWSs tracking pre-diagnosis data, in Low Middle Income Countries (LMICs), working to improve and enhance existing systems was more critical than implementing new Syndromic Surveillance approaches.

Leveraging the full capacity of Early Warning Systems to proactively detect infectious disease outbreaks is imperative more than ever before. Sources for collecting surveillance data are ample in most countries and each encompasses under-utilized pros and unforeseen cons. Nevertheless, with an exploration of EWSs functionality in different contexts and resource settings, policymakers and public health authorities should take tailored actionable steps to monitor human surveillance data and intervene at optimum times.

Many surveillance systems, whether syndromic or diagnostic disease-based, are in place in different countries globally that are run throughout the year or for mass gatherings. However, both high and LIMCs should have active surveillance to detect abnormal infectious events and utilize their primary methodology for real-time surveying pathogens. Such preparedness appears significantly urgent with the unprecedented pandemic era of current and emerging public health threats.

## Supplementary Information

**Additional file 1.** Search Strategies Appendix.

**Additional file 2.** Quality Assessment tool and Quality Assessment results table.

**Additional file 3.** Abbreviation Appendix.

## Data Availability

All the articles included in this study are available online. However, this data is available from the corresponding author upon request when possible.

## Abbreviations

ARTSSN: Alberta Real Time Syndromic Surveillance Net
CASP Checklist: Critical Appraisal Skills Programme Checklist
CBS: Community-based Surveillance
CEBS: Community Event-Based Surveillance
CHWs: Community Health Workers
CIDARS: Chinese Infectious Disease Automated-alert and Response System
COVID-19: Coronavirus disease of 2019
DF: Dengue Fever
DOHMH: Department of Health and Mental Hygiene syndromic surveillance system
EBS: Event-based Surveillance
ECDC: European Centre for Disease Prevention and Control
eDEWS: Electronic Disease Early Warning System
ED-EWS: Energency Department-Early Warning System
EDs: Emergency Departments
ED-SSS: Emergency Departments-Syndromic Surveillance System
ESSENCE: Electronic Surveillance System for the Early Notification of Community-based Epidemics
EWSs: Early Warning Systems
GP: General Practitioner
GPHIN: Global Public Health Intelligence Network
GPs: General Practitioners
HASS: Hospital Admissions Syndromic Surveillance statewide syndromic surveillance
HFM: Hand, foot, and mouth disease
HICs: High-income countries
IBS: Indicator-based Surveillance
ICARES: Integrated Crisis Alert and Response System
ICD: International Classification of Diseases
ILI: Influenza-like illness
ISIS: Infectious diseases Surveillance Information System
ISSC project: Integrated Surveillance System for infectious disease in rural China
LICs: Low-income countries
LMICs: Low Middle Income Countries
MICs: Middle-income countries
MML: Medical Microbiology Laboratories
NESOC: National Enhanced Surveillance Operations Centre
NHS: National Health Service
NHS24: National Health Service telephone helpline
NIC: National Influenza Center
NIDRIS: Notifiable Infectious Diseases Reporting Information System
OTC: Over-the-counter
OTC-EWS: Over-the-counter Early Warning System
PACES: Patient Care Enhancement System
PH: Public health
PH-EWS: Public health-Early Warning System
PICTs: Pacific island countries and territories
PRISMA: Preferred Reporting Items for Systematic Reviews and Meta-Analyses
ProMED: Program for Monitoring Emerging Diseases
PROSPERO: International Prospective Register of Systematic Reviews
RSV: Respiratory Syncytial Virus
SAGES OE: Suite for Automated Global Electronic bioSurveillance Open ESSENCE
SbSS: Syndrome-Based Surveillance System
SCM: Symptom-Clicking-Module
SIDARTHa: Spanish System for Information on Detection and Analysis of Risks and Threats to Health
SIDS: Small Island Developing States
SID-SSS: School-Based Syndromic Surveillance System
SMS: Short message service
SOS Medecins: Medical emergency service of France
SSS: Syndromic Surveillance System
SurSaUD: Reactive mortality surveillance system in France
SurvNet: Routine surveillance system in Germany
UN DESA: United Nations Department of Economic and Social Affairs
