# Supplementary material for: Effectiveness of early warning systems in the detection of infectious diseases outbreaks: a systematic review
Source: BMC Public Health. 2022 Nov 29;22:2216. doi: 10.1186/s12889-022-14625-4 (PMC9707072; doi:10.1186/s12889-022-14625-4)
Supplement: Supplementary file 1 — Additional file 1. Search Strategies Appendix. [file 12889_2022_14625_MOESM1_ESM.docx]

***Table 5. PubMed Search Strategy Appendix***

**PubMed Database**

| **Concept** | **Syntax** | **Yielded Results** | **Data** | **Links** |
| --- | --- | --- | --- | --- |
| EWS | ("Early warning system" OR Notification OR EWS OR Alert) | 68,560 | 11 September 2021 |  |
| Infectious diseases, Outbreaks | (infectious disease OR communicable diseases) AND (outbreak) | 248,892 | 11 September 2021 |  |
| Infectious diseases, Outbreaks | ("infectious disease" OR "communicable diseases") AND (outbreak) | 75,187 | 11 September 2021 |  |
| EWS | (Prevention OR Detection OR Surveillance) OR "Syndromic Surveillance" OR SSS OR "syndromic surveillance system") | 7,078,583 | 11 September 2021 |  |
| EWS, Infectious diseases, Surveillance | ((("Early warning system" OR Notification OR EWS OR Alert)) AND (("infectious disease" OR "communicable diseases") AND (outbreak))) AND ((Prevention OR Detection OR Surveillance OR "Syndromic Surveillance" OR SSS OR "syndromic surveillance system")) | 2,228 | 11 September 2021 |  |
| EWS | "Early warning system" OR Notification OR EWS OR Alert OR "syndromic surveillance" OR SSS OR "syndromic surveillance system" OR “triple S” | 75,551 | 14 September 2021 |  |
| Infectious diseases, Outbreaks | ("infectious disease" OR "communicable diseases") AND (outbreak) | 75,240 | 14 September 2021 |  |
| EWS | "Early warning system" OR Notification OR EWS OR Alert OR "syndromic surveillance" OR SSS OR "syndromic surveillance system" | 75,511 | 14 September 2021 |  |
| EWS, Infectious diseases, Outbreaks | ("Early warning system" OR Notification OR EWS OR Alert OR "syndromic surveillance" OR SSS OR "syndromic surveillance system") AND (("infectious disease" OR "communicable diseases") AND (outbreak)) | 2,441 | 14 September 2021 |  |
| EWS, Infectious diseases, Outbreaks | ("Early warning system" OR EWS OR Alert OR "syndromic surveillance" OR SSS OR "syndromic surveillance system") AND (("infectious disease" OR "communicable diseases") AND (outbreak)) | 834 | 14 September 2021 |  |
| EWS, Infectious diseases, Outbreaks | ("Early warning system" OR EWS OR Alert OR "syndromic surveillance" OR SSS OR "syndromic surveillance system") AND (("infectious disease" OR "communicable diseases") AND (outbreak)) | 836 | 16 September 2021 |  |
| **EWS, Infectious diseases, Outbreaks** | **("Early warning system" OR EWS OR Alert OR "syndromic surveillance" OR SSS OR "syndromic surveillance system") AND (("infectious disease" OR "communicable diseases") AND (outbreak))** | **947** | **21 October 2022** | <https://pubmed.ncbi.nlm.nih.gov/?term=%28%22Early+warning+system%22+OR+EWS+OR+Alert+OR+%22syndromic+surveillance%22+OR+SSS+OR+%22syndromic+surveillance+system%22%29+AND+%28%28%22infectious+disease%22+OR+%22communicable+diseases%22%29+AND+%28outbreak%29%29&sort=> |

***Table 6.* Scopus *Search Strategy Appendix***

**Scopus Database**

| **Concept** | **Syntax** | **Yielded Results** | **Data** | **Links** |
| --- | --- | --- | --- | --- |
| EWS | "Early warning system" OR ews OR alert OR "syndromic surveillance" OR sss OR "syndromic surveillance system" | 289,907 | 16 September 2021 |  |
| Infectious diseases, Outbreaks | ( "infectious disease" OR "communicable diseases" ) AND ( outbreak ) | 344,048 | 16 September 2021 |  |
| EWS, Infectious diseases, Outbreaks | ( "Early warning system" OR ews OR alert OR "syndromic surveillance" OR sss OR "syndromic surveillance system" ) AND NOT ( ( "infectious disease" OR "communicable diseases" ) AND ( outbreak ) ) | 276,288 | 16 September 2021 |  |
| EWS, Infectious diseases, Outbreaks | ( "Early warning system" OR ews OR alert OR "syndromic surveillance" OR sss OR "syndromic surveillance system" ) AND ( ( "infectious disease" OR "communicable diseases" ) AND ( outbreak ) ) | 13,619 | 16 September 2021 |  |
| EWS, Infectious diseases, Outbreaks | ( "Early warning system" OR ews OR "syndromic surveillance" OR sss OR "syndromic surveillance system" ) AND ( ( "infectious disease" OR "communicable diseases" ) AND ( outbreak ) ) | 5,167 | 16 September 2021 |  |
| EWS | "Early warning system" OR ews OR alert OR "syndromic surveillance" OR sss OR "syndromic surveillance system" | 289,907 | 16 September 2021 |  |
| EWS | ( TITLE-ABS-KEY ( "Early warning system" ) OR TITLE-ABS-KEY ( ews ) OR TITLE-ABS-KEY ( alert ) OR TITLE-ABS-KEY ( "syndromic surveillance" ) OR TITLE-ABS-KEY ( sss ) OR TITLE-ABS-KEY ( "syndromic surveillance system" ) ) | 97,811 | 16 September 2021 |  |
| Infectious diseases | ( ( TITLE-ABS-KEY ( "infectious disease" ) OR TITLE-ABS-KEY ( "communicable diseases" ) ) | 223,727 | 16 September 2021 |  |
| Outbreaks | TITLE-ABS-KEY ( outbreak ) | 194,526 | 16 September 2021 |  |
| Infectious diseases, Outbreaks | ( ( TITLE-ABS-KEY ( "infectious disease" ) OR TITLE-ABS-KEY ( "communicable diseases" ) ) ) AND ( TITLE-ABS-KEY ( outbreak ) ) | 20,319 | 16 September 2021 |  |
| EWS, Infectious diseases, Outbreaks | ( ( TITLE-ABS-KEY ( "Early warning system" ) OR TITLE-ABS-KEY ( ews ) OR TITLE-ABS-KEY ( alert ) OR TITLE-ABS-KEY ( syndromic AND surveillance ) OR TITLE-ABS-KEY ( sss ) OR TITLE-ABS-KEY ( syndromic AND surveillance AND system ) ) ) AND ( ( ( TITLE-ABS-KEY ( "infectious disease" ) OR TITLE-ABS-KEY ( "communicable diseases" ) ) ) AND ( TITLE-ABS-KEY ( outbreak ) ) ) | 654 | 16 September 2021 |  |
| EWS | ( TITLE-ABS-KEY ( "Early warning system" ) OR TITLE-ABS-KEY ( ews ) OR TITLE-ABS-KEY ( alert ) OR TITLE-ABS-KEY ( "syndromic surveillance" ) OR TITLE-ABS-KEY ( sss ) OR TITLE-ABS-KEY ( "syndromic surveillance system" ) ) | 97,406 | 16 September 2021 |  |
| EWS, Infectious diseases, Outbreaks | ( ( TITLE-ABS-KEY ( "Early warning system" ) OR TITLE-ABS-KEY ( ews ) OR TITLE-ABS-KEY ( alert ) OR TITLE-ABS-KEY ( "syndromic surveillance" ) OR TITLE-ABS-KEY ( sss ) OR TITLE-ABS-KEY ( "syndromic surveillance system" ) ) ) AND ( ( ( TITLE-ABS-KEY ( "infectious disease" ) OR TITLE-ABS-KEY ( "communicable diseases" ) ) ) AND ( TITLE-ABS-KEY ( outbreak ) ) ) | 634 | 16 September 2021 |  |
| **EWS, Infectious diseases, Outbreaks** | **( ( TITLE-ABS-KEY ( "Early warning system" ) OR TITLE-ABS-KEY ( ews ) OR TITLE-ABS-KEY ( alert ) OR TITLE-ABS-KEY ( "syndromic surveillance" ) OR TITLE-ABS-KEY ( sss ) OR TITLE-ABS-KEY ( "syndromic surveillance system" ) ) ) AND ( ( ( TITLE-ABS-KEY ( "infectious disease" ) OR TITLE-ABS-KEY ( "communicable diseases" ) ) ) AND ( TITLE-ABS-KEY ( outbreak ) ) )** | **722** | **21 October 2022** | <https://www.scopus.com/results/results.uri?sort=plf-f&src=s&sid=aeaee178b3d1033fca41dcabdb7ae6f8&sot=comb&sdt=comb&sl=329&s=%28%28TITLE-ABS-KEY+%28%22Early+warning+system%22%29+OR+TITLE-ABS-KEY+%28EWS%29+OR+TITLE-ABS-KEY+%28Alert%29+OR+TITLE-ABS-KEY+%28%22syndromic+surveillance%22%29+OR+TITLE-ABS-KEY+%28SSS%29+OR+TITLE-ABS-KEY+%28%22syndromic+surveillance+system%22%29%29%29+AND+%28%28%28TITLE-ABS-KEY+%28%22infectious+disease%22%29+OR+TITLE-ABS-KEY+%28%22communicable+diseases%22%29%29%29+AND+%28TITLE-ABS-KEY+%28outbreak%29%29%29&origin=savedSearchNewOnly&txGid=b108e4c7c4d459b69324744618b5861e> |
