# Supplementary material for: Effectiveness of early warning systems in the detection of infectious diseases outbreaks: a systematic review
Source: BMC Public Health. 2022 Nov 29;22:2216. doi: 10.1186/s12889-022-14625-4 (PMC9707072; doi:10.1186/s12889-022-14625-4)
Supplement: Supplementary file 2 — Additional file 2. Quality Assessment tool and Quality Assessment results table. [file 12889_2022_14625_MOESM2_ESM.docx]

***Table 2: Quality Assessment Tool***

| Is there a clear statement of the aim of the research? (Y/N) |
| --- |
| Is the test data set reproducible? (Y/N) |
| Is it clearly stated in the study which other algorithms the study's algorithms have been compared with? (Y/N) |
| Was the methodology of evaluation and/or EWSs functioning process described? (Y/N) |
| Are the test results thoroughly analyzed? EWS sensitivity/specificity, EWS effectiveness as an early predictor, pros and cons of the system (Y/N) |
| Did the study have limitations or risk of bias mentioned by the authors? (Y/N) |

***Table 3: Quality Assessment Results***

| **Author and Year** | **Is there a clear statement of the aim of the research? (Y=1/N=0)** | **Is the test data set reproducible? (Y=1/N=0)** | **Is it clearly stated in the study which other algorithms the study's algorithms have been compared with? (Y=1/N=0)** | **Was the methodology of evaluation and/or EWSs functioning process described? (Y=1/N=0)** | **Are the test results thoroughly analyzed? EWS sensitivity/specificity, EWS effectiveness as an early predictor, pros and cons of the system (Y=1/N=0)** | **Did the study have limitations or risk of bias mentioned by the authors? (Y=1/N=0)** | **QA Score out of 6** |
| --- | --- | --- | --- | --- | --- | --- | --- |
| Mostashari, F. et al.,2003 | 0 | 1 | 0 | 1 | 1 | 1 | 4 |
| Schrell, S. et al.,2013 | 0 | 1 | 1 | 1 | 1 | 1 | 5 |
| Das, D. et al.,2015 | 1 | 1 | 1 | 1 | 1 | 1 | 6 |
| Dong, X.et al.,2017 | 1 | 1 | 1 | 1 | 1 | 1 | 6 |
| Ansaldi, F.et al.,2009 | 1 | 1 | 1 | 1 | 1 | 1 | 6 |
| Cashmore, A. W. et al.,2013 | 1 | 1 | 0 | 1 | 1 | 1 | 5 |
| Elliot, A. J. et al.,2012 | 1 | 1 | 0 | 1 | 1 | 1 | 5 |
| Hope, K. G. et al., 2010 | 0 | 1 | 1 | 1 | 1 | 1 | 5 |
| Lall, R. et al.,2017 | 1 | 1 | 1 | 1 | 1 | 1 | 6 |
| Muscatello, D. J. et al., 2005 | 0 | 1 | 0 | 1 | 1 | 1 | 4 |
| Terry, W. et al., 2004 | 1 | 1 | 1 | 1 | 1 | 1 | 6 |
| Tsung-Shu Joseph Wu et al., 2008 | 0 | 1 | 0 | 1 | 1 | 1 | 4 |
| Ansaldi, F. et al., 2008 | 1 | 1 | 1 | 1 | 1 | 1 | 6 |
| Schenkel, K. et al., 2006 | 1 | 1 | 1 | 1 | 1 | 1 | 6 |
| White, P. et al., 2017 | 1 | 1 | 0 | 1 | 1 | 0 | 4 |
| Paul White et al., 2018 | 0 | 1 | 0 | 1 | 1 | 0 | 3 |
| C. J. WILLIAMS,K. et al., 2009 | 1 | 1 | 1 | 1 | 1 | 1 | 6 |
| Ye, C. et al., 2016 | 1 | 1 | 1 | 1 | 1 | 1 | 6 |
| Buda, S. et al., 2017 | 1 | 1 | 1 | 1 | 1 | 1 | 6 |
| Dembek, Z. F. et al., 2004 | 1 | 1 | 1 | 1 | 1 | 1 | 6 |
| Zeldenrust, M. E. et al., 2008 | 0 | 1 | 0 | 1 | 1 | 1 | 4 |
| Witkop et al., 2009 | 1 | 1 | 0 | 1 | 1 | 1 | 5 |
| Betancourt, J. A. et al., 2007 | 1 | 1 | 1 | 1 | 1 | 1 | 6 |
| Carneiro, H. A. and Mylonakis, E., 2009 | 1 | 1 | 0 | 1 | 1 | 1 | 5 |
| Dion, M. et al., 2015 | 1 | 1 | 0 | 1 | 1 | 0 | 4 |
| Samaras, L. et al., 2021 | 1 | 1 | 1 | 1 | 1 | 1 | 6 |
| van Benthem, B. H. and van Vliet, J. A., 2008 | 0 | 1 | 0 | 1 | 1 | 1 | 4 |
| Dureab, F. et al., 2020 | 1 | 1 | 0 | 1 | 1 | 1 | 5 |
| Fan, S. et al., 2010 | 1 | 1 | 1 | 1 | 1 | 1 | 6 |
| Lowe, R. et al., 2016 | 0 | 1 | 1 | 1 | 1 | 1 | 5 |
| Nuvey, F. S. et al., 2019 | 1 | 1 | 0 | 1 | 1 | 1 | 5 |
| Yang, W. et al., 2011 | 1 | 1 | 1 | 1 | 1 | 1 | 6 |
| Zhang, H. et al., 2014 | 0 | 1 | 0 | 1 | 1 | 1 | 4 |
| Aggrawal et al., 2020 | 1 | 1 | 0 | 1 | 1 | 1 | 5 |
| Ang, B. C. et al., 2005 | 1 | 1 | 0 | 1 | 1 | 1 | 5 |
| Baghdadi, Y. et al., 2019 | 1 | 1 | 0 | 1 | 1 | 0 | 4 |
| Kool, J. L. et al., 2012 | 0 | 1 | 0 | 1 | 1 | 1 | 4 |
| Bijkerk, P. et al., 2017 | 1 | 1 | 0 | 1 | 0 | 1 | 4 |
| van den Wijngaard, C. et al., 2008 | 0 | 1 | 0 | 1 | 1 | 1 | 4 |
| van den Wijngaard, C. C. et al., 2010 | 1 | 1 | 1 | 1 | 1 | 0 | 5 |
| Weirong Yan et al., 2013 | 1 | 1 | 1 | 1 | 1 | 1 | 6 |
| El-Khatib, Z. et al., 2019 | 1 | 1 | 0 | 1 | 1 | 1 | 5 |
| Flamand, C. et al., 2008 | 1 | 1 | 1 | 1 | 1 | 1 | 6 |
| Jones, N. F. and Marshall, R., 2004 | 1 | 1 | 0 | 1 | 1 | 0 | 4 |
| Merali, S. et al., 2020 | 0 | 0 | 0 | 1 | 1 | 1 | 3 |
| Murray, K. O. et al., 2009 | 0 | 1 | 0 | 1 | 1 | 0 | 3 |
| Randrianasolo, L. et al., 2010 | 0 | 1 | 1 | 1 | 1 | 1 | 5 |
| Ratnayake, R. et al., 2016 | 1 | 1 | 0 | 1 | 1 | 1 | 5 |
| Weng, T. C. et al., 2015 | 1 | 1 | 0 | 1 | 1 | 1 | 5 |
| Groeneveld, G. H. et al., 2017 | 1 | 1 | 1 | 1 | 1 | 1 | 6 |
| Caudle, J. M. et al., 2009 | 1 | 1 | 0 | 1 | 1 | 1 | 5 |
| Cooper, D. L. et al., 2006 | 1 | 1 | 1 | 1 | 1 | 1 | 6 |
| Doroshenko, A. et al., 2005 | 1 | 1 | 1 | 1 | 1 | 1 | 6 |
| Dureab, F.et al., 2019 | 1 | 1 | 0 | 1 | 1 | 1 | 5 |
| Katayama, Y. et al., 2020 | 1 | 1 | 0 | 1 | 1 | 1 | 5 |
| Katayama, Y. et al., 2021 | 1 | 1 | 0 | 1 | 1 | 1 | 5 |
| Kavanagh, K. et al., 2012 | 0 | 1 | 0 | 1 | 1 | 1 | 4 |
| Guzman-Herrador, B. et al., 2016 | 1 | 1 | 1 | 1 | 1 | 1 | 6 |
| Li, Z. et al., 2014 | 1 | 1 | 0 | 1 | 1 | 0 | 4 |
| Pinto, A. et al., 2005 | 1 | 1 | 0 | 1 | 1 | 1 | 5 |
| Stikova, E. et al., 2010 | 0 | 0 | 1 | 0 | 0 | 0 | 1 |
| Ganeshkumar et al., 2022 | 1 | 1 | 0 | 1 | 1 | 1 | 5 |
| Hong et al., 2022 | 1 | 1 | 1 | 1 | 1 | 1 | 6 |
| Lai et al., 2021 | 1 | 1 | 0 | 1 | 1 | 1 | 5 |
| Lami et al. 2021 | 1 | 1 | 1 | 1 | 1 | 1 | 6 |
| Leining et al., 2022 | 1 | 1 | 1 | 1 | 1 | 1 | 6 |
| Lukowsky et al., 2022 | 0 | 1 | 0 | 1 | 1 | 1 | 4 |
| Yang et al., 2022 | 1 | 1 | 1 | 1 | 1 | 1 | 6 |
