# Supplementary material for: Effectiveness of early warning systems in the detection of infectious diseases outbreaks: a systematic review
Source: BMC Public Health. 2022 Nov 29;22:2216. doi: 10.1186/s12889-022-14625-4 (PMC9707072; doi:10.1186/s12889-022-14625-4)
Supplement: Supplementary file 3 — Additional file 3. Abbreviation Appendix. [file 12889_2022_14625_MOESM3_ESM.docx]

***Table 4. Abbreviation Appendix***

| **Abbreviation** | **Meaning** |
| --- | --- |
| ARTSSN | Alberta Real Time Syndromic Surveillance Net |
| CASP Checklist | Critical Appraisal Skills Programme Checklist |
| CBS | Community-based surveillance |
| CEBS | Community Event-Based Surveillance |
| CHWs | Community Health Workers |
| CIDARS | Chinese Infectious Disease Automated-alert and Response System |
| COVID-19 | Coronavirus disease of 2019 |
| DF | Dengue Fever |
| DOHMH | Department of Health and Mental Hygiene syndromic surveillance system |
| EBS | event-based surveillance |
| ECDC | European Centre for Disease Prevention and Control |
| eDEWS | Electronic Disease Early Warning System |
| ED-EWS | Energency Department-Early Warning System |
| EDs | Emergency Departments |
| ED-SSS | Emergency Departments-Syndromic Surveillance System |
| ESSENCE | Electronic Surveillance System for the Early Notification of Community-based Epidemics |
| EWSs | Early Warning Systems |
| GP | General Practitioner |
| GPHIN | Global Public Health Intelligence Network |
| GPs | General Practitioners |
| HASS | Hospital Admissions Syndromic Surveillance statewide syndromic surveillance |
| HFM | Hand, foot, and mouth disease |
| HICs | high-income countries |
| IBS | indicator-based surveillance |
| ICARES | Integrated Crisis Alert and Response System |
| ICD | International Classification of Diseases |
| ILI | influenza-like illness |
| ISIS | Infectious diseases Surveillance Information System |
| ISSC project | Integrated Surveillance System for infectious disease in rural China |
| LICs | low-income countries |
| LMICs | Low Middle Income Countries |
| MICs | middle-income countries |
| MML | Medical Microbiology Laboratories |
| NESOC | National Enhanced Surveillance Operations Centre |
| NHS | National Health Service |
| NHS24 | National Health Service telephone helpline |
| NIC | National Influenza Center |
| NIDRIS | Notifiable Infectious Diseases Reporting Information System |
| OTC | over-the-counter |
| OTC-EWS | over-the-counter Early Warning System |
| PACES | Patient Care Enhancement System |
| PH | Public health |
| PH-EWS | Public health-Early Warning System |
| PICTs | Pacific island countries and territories |
| PRISMA | Preferred Reporting Items for Systematic Reviews and Meta-Analyses |
| ProMED | Program for Monitoring Emerging Diseases |
| PROSPERO | International Prospective Register of Systematic Reviews |
| RSV | Respiratory Syncytial Virus |
| SAGES OE | Suite for Automated Global Electronic bioSurveillance Open ESSENCE |
| SbSS | Syndrome-Based Surveillance System |
| SCM | Symptom-Clicking-Module |
| SIDARTHa | Spanish System for Information on Detection and Analysis of Risks and Threats to Health |
| SIDS | Small Island Developing States |
| SID-SSS | School-Based Syndromic Surveillance System |
| SMS | short message service |
| SOS Medecins | medical emergency service of France |
| SSS | Syndromic Surveillance System |
| SurSaUD | Reactive mortality surveillance system in France |
| SurvNet | routine surveillance system in Germany |
| UN DESA | United Nations Department of Economic and Social Affairs |
